# Effects of a comprehensive brain computed tomography deep learning model on radiologist detection accuracy

**DOI:** 10.1007/s00330-023-10074-8

**Published:** 2023-08-22

**Authors:** Quinlan D. Buchlak, Cyril H. M. Tang, Jarrel C. Y. Seah, Andrew Johnson, Xavier Holt, Georgina M. Bottrell, Jeffrey B. Wardman, Gihan Samarasinghe, Leonardo Dos Santos Pinheiro, Hongze Xia, Hassan K. Ahmad, Hung Pham, Jason I. Chiang, Nalan Ektas, Michael R. Milne, Christopher H. Y. Chiu, Ben Hachey, Melissa K. Ryan, Benjamin P. Johnston, Nazanin Esmaili, Christine Bennett, Tony Goldschlager, Jonathan Hall, Duc Tan Vo, Lauren Oakden-Rayner, Jean-Christophe Leveque, Farrokh Farrokhi, Richard G. Abramson, Catherine M. Jones, Simon Edelstein, Peter Brotchie

**Affiliations:** 1 Annalise.ai, Sydney, NSW Australia; 2 School of Medicine, University of Notre Dame Australia, Sydney, NSW Australia; 3 Department of Neurosurgery, Monash Health, Clayton, VIC Australia; 4 Department of Radiology, Alfred Health, Melbourne, VIC Australia; 5 Department of Radiology, University Medical Center, University of Medicine and Pharmacy, Ho Chi Minh City, Vietnam; 6 Department of General Practice, University of Melbourne, Melbourne, VIC Australia; 7 Westmead Applied Research Centre, University of Sydney, Sydney, NSW Australia; 8 Faculty of Engineering and Information Technology, University of Technology Sydney, Sydney, NSW Australia; 9 Department of Surgery, Monash University, Clayton, VIC Australia; 10 Department of Radiology, St Vincent’s Health Australia, Melbourne, VIC Australia; 11 Department of Radiology, Austin Hospital, Melbourne, VIC Australia; 12 Australian Institute for Machine Learning, The University of Adelaide, Adelaide, SA Australia; 13 Center for Neurosciences and Spine, Virginia Mason Franciscan Health, Seattle, WA USA; 14 I-MED Radiology Network, Brisbane, QLD Australia; 15 School of Public and Preventive Health, Monash University, Clayton, VIC Australia; 16 Department of Clinical Imaging Science, University of Sydney, Sydney, NSW Australia; 17 Department of Radiology, Monash Health, Clayton, VIC Australia

**Keywords:** Machine learning, Supervised machine learning, Tomography, x-ray computed, Brain, Artificial intelligence

## Abstract

**Objectives:**

Non-contrast computed tomography of the brain (NCCTB) is commonly used to detect intracranial pathology but is subject to interpretation errors. Machine learning can augment clinical decision-making and improve NCCTB scan interpretation. This retrospective detection accuracy study assessed the performance of radiologists assisted by a deep learning model and compared the standalone performance of the model with that of unassisted radiologists.

**Methods:**

A deep learning model was trained on 212,484 NCCTB scans drawn from a private radiology group in Australia. Scans from inpatient, outpatient, and emergency settings were included. Scan inclusion criteria were age ≥ 18 years and series slice thickness ≤ 1.5 mm. Thirty-two radiologists reviewed 2848 scans with and without the assistance of the deep learning system and rated their confidence in the presence of each finding using a 7-point scale. Differences in AUC and Matthews correlation coefficient (MCC) were calculated using a ground-truth gold standard.

**Results:**

The model demonstrated an average area under the receiver operating characteristic curve (AUC) of 0.93 across 144 NCCTB findings and significantly improved radiologist interpretation performance. Assisted and unassisted radiologists demonstrated an average AUC of 0.79 and 0.73 across 22 grouped parent findings and 0.72 and 0.68 across 189 child findings, respectively. When assisted by the model, radiologist AUC was significantly improved for 91 findings (158 findings were non-inferior), and reading time was significantly reduced.

**Conclusions:**

The assistance of a comprehensive deep learning model significantly improved radiologist detection accuracy across a wide range of clinical findings and demonstrated the potential to improve NCCTB interpretation.

**Clinical relevance statement:**

This study evaluated a comprehensive CT brain deep learning model, which performed strongly, improved the performance of radiologists, and reduced interpretation time. The model may reduce errors, improve efficiency, facilitate triage, and better enable the delivery of timely patient care.

**Key Points:**

*• This study demonstrated that the use of a comprehensive deep learning system assisted radiologists in the detection of a wide range of abnormalities on non-contrast brain computed tomography scans.*

*• The deep learning model demonstrated an average area under the receiver operating characteristic curve of 0.93 across 144 findings and significantly improved radiologist interpretation performance.*

*• The assistance of the comprehensive deep learning model significantly reduced the time required for radiologists to interpret computed tomography scans of the brain.*

**Supplementary Information:**

The online version contains supplementary material available at 10.1007/s00330-023-10074-8.

## Introduction

Computed tomography (CT), invented in the 1970s, was the first method available for direct imaging of the brain and is still the primary imaging modality used for this purpose. Non-contrast computed tomography of the brain (NCCTB) is commonly used for patients with suspected intracranial pathology, primarily due to its accessibility and short acquisition time [1]. In emergency medicine, NCCTB enables rapid diagnosis and the provision of timely care to patients who might otherwise suffer substantial morbidity or mortality [1, 2]. Over 15 million NCCTB studies were conducted in 2016 in the USA [3]. Even amongst expert radiologist readers, error patterns have been reported for infarct detection, extra-axial masses, and vessel thrombosis [4, 5], with clinician inexperience, fatigue, and interruptions appearing to increase error likelihood [6]. To address these issues, attempts have been made to develop artificial intelligence (AI) systems to mitigate errors and assist clinicians with interpretation [7].

Deep learning convolutional neural networks (CNNs) are a class of neural network designed to process multi-dimensional image data. CNNs have been applied successfully to many domains of medicine [8, 9] and have demonstrated strong image classification performance in radiology [10, 11]. Deep learning systems in radiology appear to improve the clinical finding detection ability of radiologists [10], particularly junior clinicians [11], and have facilitated reductions in mean interpretation time [10, 12]. Most NCCTB deep learning systems developed, however, have been limited in scope, capable of detecting just a single or a small number of clinical findings. Chilamkurthy et al (2018) trained and validated a model that could accurately detect four critical clinical findings (including multiple haemorrhage types), using a dataset consisting of 313,318 NCCTBs automatically labelled using radiology reports [7]. Other deep learning systems have been developed to accurately detect intracranial haemorrhage [13], traumatic brain injury [14], acute infarction [15], and dementia [16]. However, the narrow scope of extant systems limits their clinical utility. There is a trend toward increasing the clinical comprehensiveness of deep learning systems for other modalities [10, 17] and considerable opportunity exists to improve the scope of deep learning systems designed to facilitate NCCTB interpretation.

We developed and evaluated a comprehensive deep learning system designed to assist clinicians with the interpretation of NCCTB studies and provide notification of suspected findings. The system is indicated for use with non-contrast brain CT scans (brain kernel) of adult patients. Research questions included the following: (1) How does radiologist interpretation performance change when the deep learning system is used as an assistant? (2) How does the comprehensive deep learning model perform in comparison to experienced practising radiologists?

## Method

### Study design

A retrospective multi-reader multi-case (MRMC) study was designed to evaluate the detection accuracy of 32 radiologists with and without the aid of the deep learning system. Radiologists first interpreted cases without access to the deep learning tool, and then re-interpreted the same set of cases with assistance from the deep learning tool following a minimum 4-month (124-day) wash-out period.

Model development and evaluation involved NCCTB dataset labelling and interpretation by three mutually exclusive groups of radiologists performing distinct functions:

1. Initial classification labelling of the wider dataset that included both test and training data was performed by 143 consultant radiologists from Vietnam,
2. Dawid-Skene consensus of the labels on the test dataset was calculated and ground-truth adjudication of these labels was performed by three specialist neuroradiologists from Australia, and
3. Interpretation of the test dataset in the MRMC study was performed by 32 consultant radiologists from Vietnam.

Classification labelling of the wider dataset identified the radiological findings present on each case, as defined by an ontology tree prospectively developed by consultant neuroradiologists that contained 214 clinical findings (192 child findings and 22 parents; Supplementary Materials). Ground-truth labelling identified the radiological findings present in the test dataset cases used in this MRMC study.

### Ethics approvals

This study was approved by the Bellberry Human Research Ethics Committee (HREC; approval numbers: 2021–02-123 and 2021–03-259), the University of Notre Dame Australia’s HREC (approval number: 2020-127S), and the University of Medicine and Pharmacy at Ho Chi Minh City’s Board of Ethics in Biomedical Research (IRB-VNO1002). A waiver of consent for use of the de-identified CT data in this study was approved with consideration of Australia’s National Statement of Ethical Conduct in Human Research.

### Ontology tree

An ontology tree was developed, specifying clinical findings and describing relationships between these findings (Supplementary Materials). Each of the 214 findings was defined by a consensus of three Australian subspecialist neuroradiologists. Radiologists engaged in labelling and evaluation were trained to identify the NCCTB findings according to these definitions.

### Data

This study involved the use of 215,332 NCCTBs, from 170,745 unique patients, which were drawn from a private radiology group in Australia. Cases included scans from inpatient, outpatient, and emergency settings. Inclusion criteria were age ≥ 18 years and series slice thickness ≤ 1.5 mm. NCCTBs underwent classification labelling for each child finding of the ontology tree and each was labelled by three to eight radiologists. Labellers completed training prior to commencing, which involved familiarisation with the annotation tool, reviewing the definitions of each finding, and practice on a curated dataset of 183 NCCTBs. Labeller performance was assessed with the F1 metric [18] and each demonstrated an F1 score > 0.50 before commencement. Each radiologist was given the same data for each case but was blinded to labels generated by the other radiologists. The radiology report, patient age, and sex were provided, along with all series in the study, and paired CT or magnetic resonance imaging (MRI) scans. A consensus classification label for each finding in each case was generated as a score between 0 and 1 using the Dawid-Skene algorithm [19]. Localisation labelling (3D segmentation and lateralisation) was performed for a subset of findings (Supplementary Materials). Labellers were provided with the positive localizable findings and were instructed to segment/lateralize only those findings. Segmentation maps were each labelled by three radiologists.

### Training dataset

A subset of the data, comprising 212,484 NCCTBs (168,326 unique patients), was used for training. Classification labels were used to train the model to detect findings. Parent findings were automatically labelled based on child labels. The model learned from the original labels and the structure of the ontology tree. The segmented maps were used to train the model to produce overlay outputs.

### Test dataset

A power analysis determined that a minimum MRMC test dataset of 2848 cases (2419 unique patients) was required to detect a mean difference in area under the receiver operating characteristic curve (AUC) of 0.02 in the detection accuracy of 30 radiologists (alpha = 0.05, beta = 0.8). Cases were drawn from the labelled dataset to achieve a sufficient number of cases per finding while keeping the total number of cases as low as possible. MRMC test dataset cases were excluded from model training at the patient level. Each case in the test dataset underwent an adjudicated ground-truth labelling process to ensure a high-quality gold standard. Ground-truth labels were determined by one of three fellowship-trained subspecialist neuroradiologists who reviewed the Dawid-Skene consensus labels and the classification labels chosen by the initial three labellers. These neuroradiologists had access to anonymized clinical information, past and future radiological investigations, and radiology reports. They did not have access to the outputs of the deep learning model.

### Deep learning model development

The deep learning model consisted of an ensemble of five CNNs trained using fivefold cross-validation. The model had three heads: one for classification, one for left–right localisation, and one for segmentation. Models were based on the ResNet [20], Y-Net [21], and ViT [22] architectures. A single ensemble model was trained on all findings simultaneously. Class imbalance was mitigated using class-balanced loss weighting and super-sampling of instances with segmentation labels. Study endpoints addressed the performance of the classification model (v1.0). A total of 144 findings were selected for inclusion in the AI model during the MRMC study based on clinical and statistical considerations during model development. Included findings were required to (1) achieve an AUC of at least 0.80; (2) be able to achieve a minimum precision of 0.20 at the chosen operating threshold; (3) have at least 50 cases in the training set; and (4) demonstrate performance that was not lower than previously published AI performance for comparable clinical findings. Beta values were chosen by the team of subspecialist neuroradiologists based on the criticality of the finding. The higher the criticality, the less tolerance for missing a finding and thus a higher beta was chosen to improve the sensitivity of the model.

### MRMC test dataset interpretation

Thirty-two radiologists, each with 2 to 21 years of clinical experience after completion of radiology specialist training (median = 8 years), each interpreted all 2848 cases in the MRMC dataset. Patient age, sex, and the clinical stem of the radiology request were shown but no radiological report or other comparison images were provided. Radiologists were asked to rate their confidence in the presence of each of the 214 findings in the ontology tree using a 7-point scale. The AI tool displayed findings detected by the deep learning model, along with a measure of the model’s confidence. For a subset of findings, a segmentation overlay was displayed. Radiologist interaction was performed on diagnostic-quality monitors and hardware. Interpretation times were recorded by the DICOM viewing platform. Radiologists were trained on ontology tree definitions and the labelling methodology. They then independently evaluated all 2848 studies without model assistance. After a wash-out period, training on use of the AI tool was provided, and the same radiologists independently evaluated the studies again with model assistance.

### Analysis

The primary objective of this study was to quantify the difference in radiologist detection performance with and without assistance from the model. The secondary objective was to compare the performance metrics of unassisted readers with the standalone deep learning model. For the primary objective, differences in AUC and Matthews correlation coefficient (MCC) were calculated. AUC and MCC were reported as primary metrics because AUC is a widely accepted machine learning performance metric and the MCC provides a more informative indication of classifier performance than other metrics. Receiver operating characteristic (ROC) curves were plotted; US Food and Drug Administration (FDA) iMRMC v4.0.3 software and the generalized Roe and Metz model were used to analyse radiologist performance (AUCs) with and without assistance from the model [23, 24]. Bootstrapping was used to determine if there was a statistically significant difference in average radiologist performance for each finding between arms. The Benjamini–Hochberg procedure (alpha = 0.05) was used to control the false discovery rate accounting for multiple comparisons [25]. A difference in AUC greater than 0.05 was considered clinically significant [26]; clinical non-inferiority was defined as the lower tail of the two-sided 95% CI being greater than − 0.05, and clinical inferiority was defined as the upper tail of the two-sided 95% CI being less than − 0.05. Statistical superiority was defined as the lower tail of the two-sided 95% CI being greater than zero and statistical inferiority was defined as the upper tail being less than zero [10]. A clinically significant MCC was considered as a difference greater than 0.1. For the secondary objective, the AUC of the model was compared to the average unassisted radiologist AUC for each finding using the same bootstrapping technique. Analyses were conducted and verified by multiple researchers (C.T., J.S., L.D.S.P.). The methodology was verified by an independent professor of biostatistics (G.H.).

## Results

Training and test dataset characteristics are outlined in Table 1. Model assistance improved radiologist interpretation performance. Unassisted and assisted radiologists demonstrated an average AUC of 0.73 and 0.79 across the 22 parent findings, respectively. Three child findings had too few cases to calculate reader performance (“enlarged vestibular aqueduct”: 0, “intracranial pressure monitor”: 0, and “longus colli calcification”: 1). Unassisted radiologists demonstrated an average AUC of 0.68 across the remaining 189 child findings. The lowest AUC was obtained for “intraventricular debris” (0.50, 95% CI = 0.49–0.51). The highest AUCs were obtained for “deep brain stimulation (DBS) electrodes” (0.97, 95% CI = 0.95–0.99), “ventriculoperitoneal (VP) shunt” (0.96, 95% CI = 0.95–0.97), and “aneurysm coils” (0.95, 95% CI = 0.93–0.98). Assisted radiologists demonstrated an average AUC of 0.72 across the 189 child findings. The lowest AUC was obtained for “intraventricular debris” (0.50, 95% CI = 0.500.50). The highest AUCs were obtained for “DBS electrodes” (0.99, 95% CI = 0.99–1.00), “aneurysm coils” (0.97, 95% CI = 0.94–0.99) and “VP shunt” (0.97, 95% CI = 0.95–0.98).

**Table 1:** Training and testing (i.e. MRMC) dataset details. Data are displayed as *n* (%), mean (SD), or median (IQR)

	Training dataset	Testing dataset
Studies	212,484	2848
Patients	168,326	2419
Sex		
Male	90,299 (53.6%)	1292 (53.4%)
Female	77,911 (46.3%)	1125 (46.5%)
Unknown/other	116 (0.1%)	2 (0.1%)
Mean age, years	66.9 y (SD 18.5 y)	64.4y (SD 18.2 y)
Median number of findings per study	4 (IQR 2–6)	7 (IQR 4–10)

**Fig. 1:**
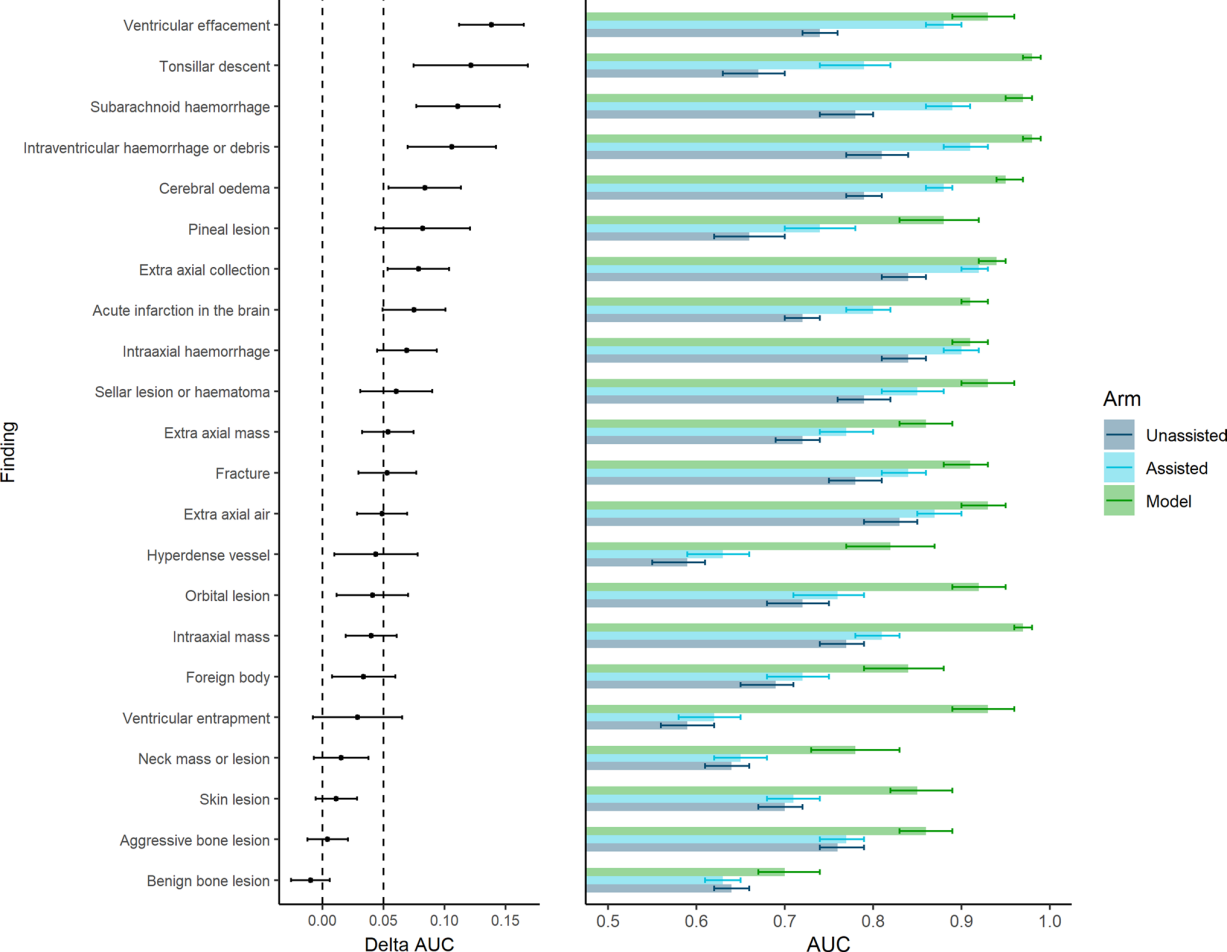
Change in AUC of parent findings when radiologists were assisted by the deep learning model. Mean AUCs of the model, unassisted, and assisted radiologists and change in (i.e. delta) AUC, along with adjusted 95% CIs, are shown for each parent finding. Findings were considered clinically significant where the lower limit of the 95% CI was greater than 0.05, and statistically significant where the lower limit of the 95% CI was greater than zero

Change in radiologist AUC when assisted by the model was positive and statistically significant for 91 child findings. The three findings that demonstrated the largest AUC increase were “uncal herniation” (AUC increase 0.19, 95% CI = 0.14–0.24), “sulcal effacement” (AUC increase 0.19, 95% CI = 0.16–0.21), and “tonsillar herniation” (AUC increase 0.19, 95% CI = 0.12–0.25). Seventeen AUC decrements were identified when the model was used as an assistant, sixteen of which were statistically inferior. One hundred and fifty-eight findings were clinically non-inferior and only one statistically significant decrement was clinically significant (“cerebellar agenesis”). Figure 1 presents assisted and unassisted radiologist AUCs for the 22 parent findings. All statistics are presented in Supplementary Materials.

Model use was associated with a statistically significantly lower mean interpretation time (26.5 s faster with model assistance, 95% CI = 13–41 s, *p* < 0.01). The mean interpretation time in study arm one was 236.0 s (median 198.0 s, IQR 140.8–282.2 s), whereas the mean interpretation time in arm two was 209.5 s (median 163.5 s, IQR 106.5–254.8 s).

Eighty-one child findings demonstrated a statistically significant improvement in MCC when radiologists used the deep learning model as an assistant. One hundred and sixty-nine child findings were clinically non-inferior (lower tail of the ∆MCC 95% CI greater than − 0.1). There was one statistically inferior finding (“cerebellar agenesis”).

### Standalone model performance

**Fig. 2:**
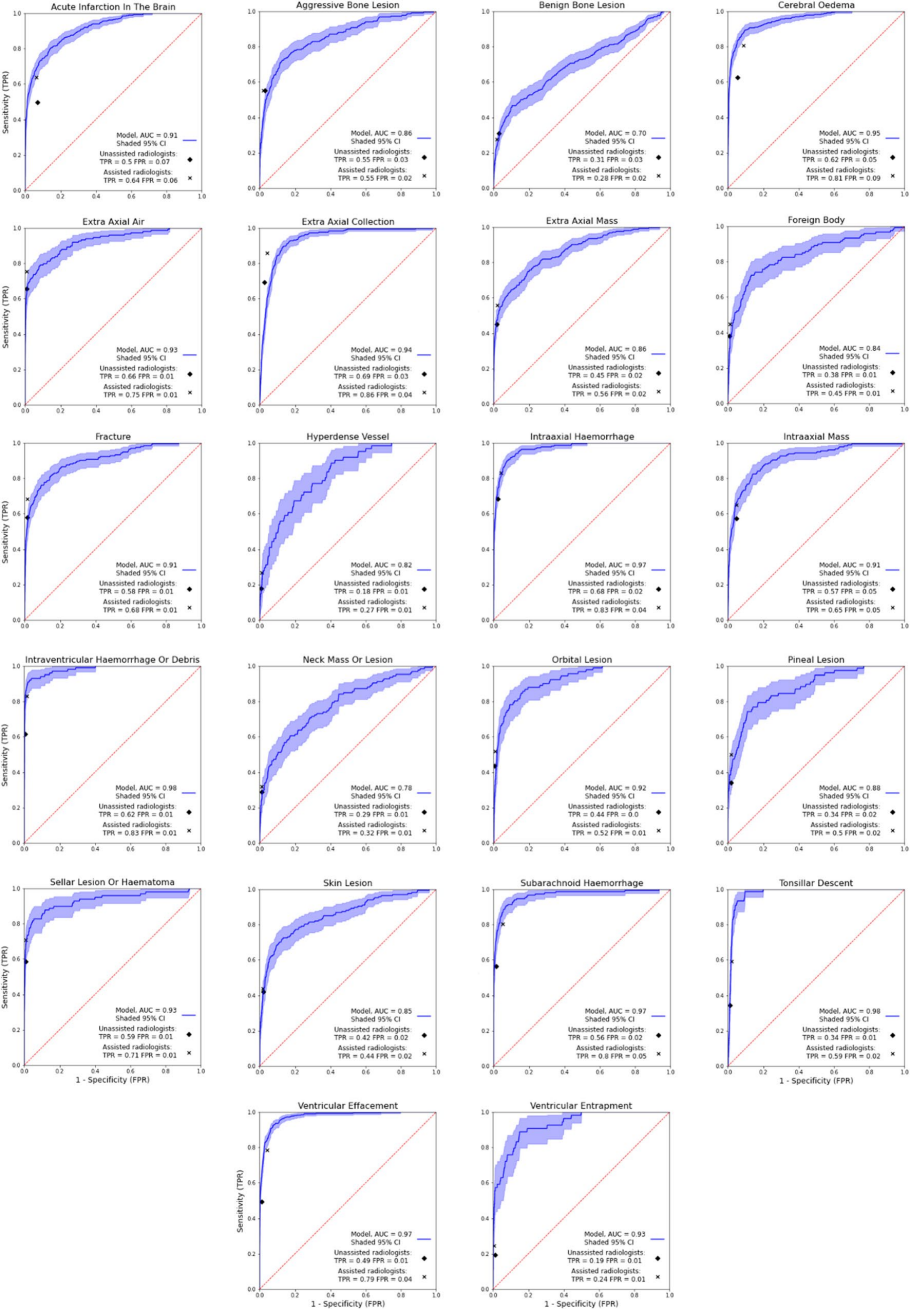
ROC curves for the parent findings demonstrating the performance of the model, and the mean performance of the assisted and unassisted radiologists

Forty-eight findings were excluded from the final model due to inadequate performance (Supplementary Materials), resulting in a total of 144 model findings. The model alone demonstrated an average AUC of 0.93 across all 144 model findings and 0.90 across the parent findings. Lowest AUCs were obtained for “non-aggressive bone lesion” (0.74, 95% CI 0.68–0.80) and “non-aggressive extra-axial fat density” (0.74, 95% CI 0.63–0.85). The highest AUCs of 1.00 were obtained for “DBS electrodes” (95% CI 1.00–1.00), and “cochlear implant” (95% CI 1.00–1.00). Model AUC was statistically superior to unassisted radiologist performance for 142 clinical findings. The two remaining findings were inconclusive. ROC curves comparing the performance of the model with the mean performance of radiologists are presented in Fig. 2 (parent findings).

**Fig. 3:**
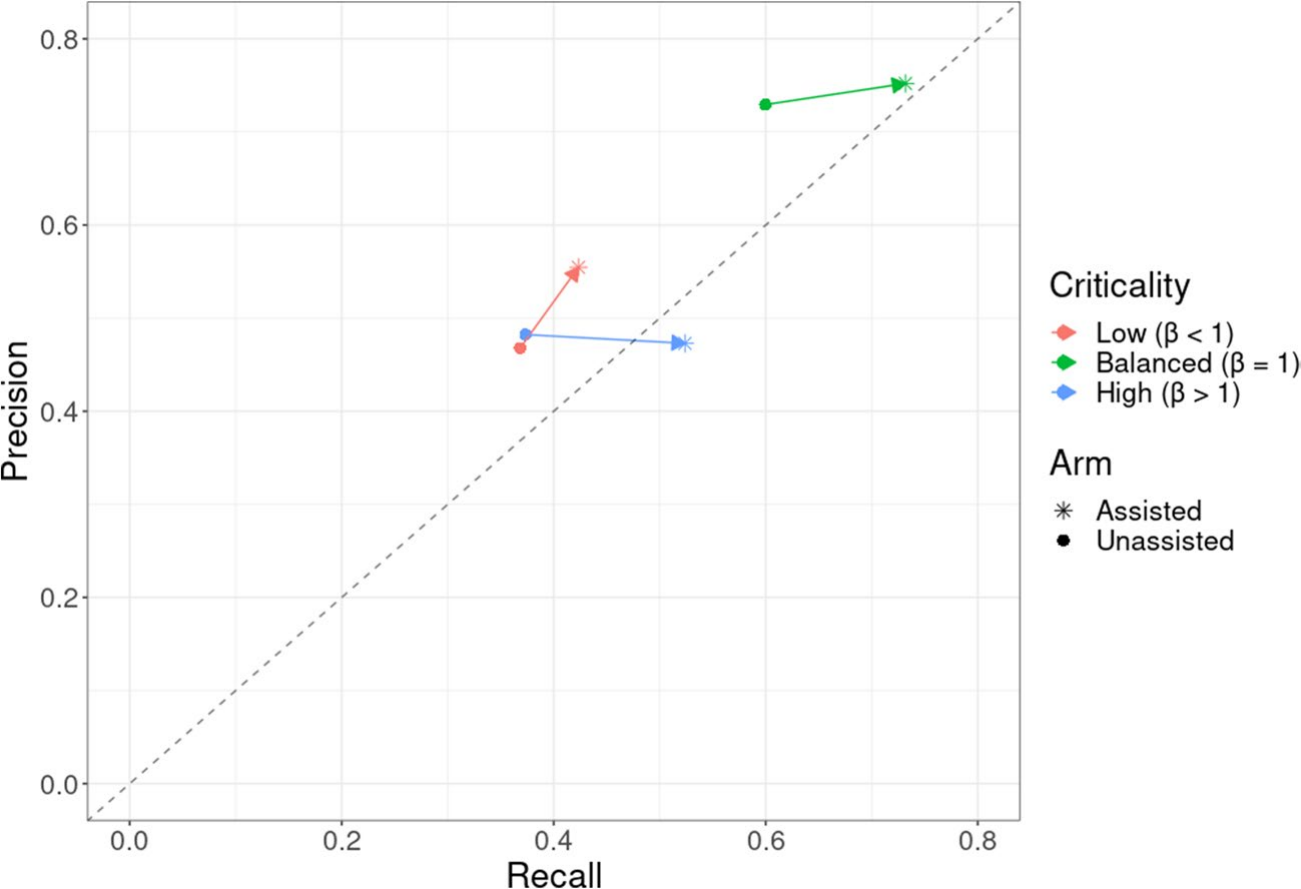
Performance improvement using the deep learning model. Precision and recall (i.e. sensitivity) for the unassisted and assisted radiologists averaged across all findings, based on the chosen beta levels for each finding. Arrows indicate the shift in recall and precision of the radiologists when assisted by AI. On average, model assistance resulted in increased recall (sensitivity) with no decrement in precision

**Fig. 4:**
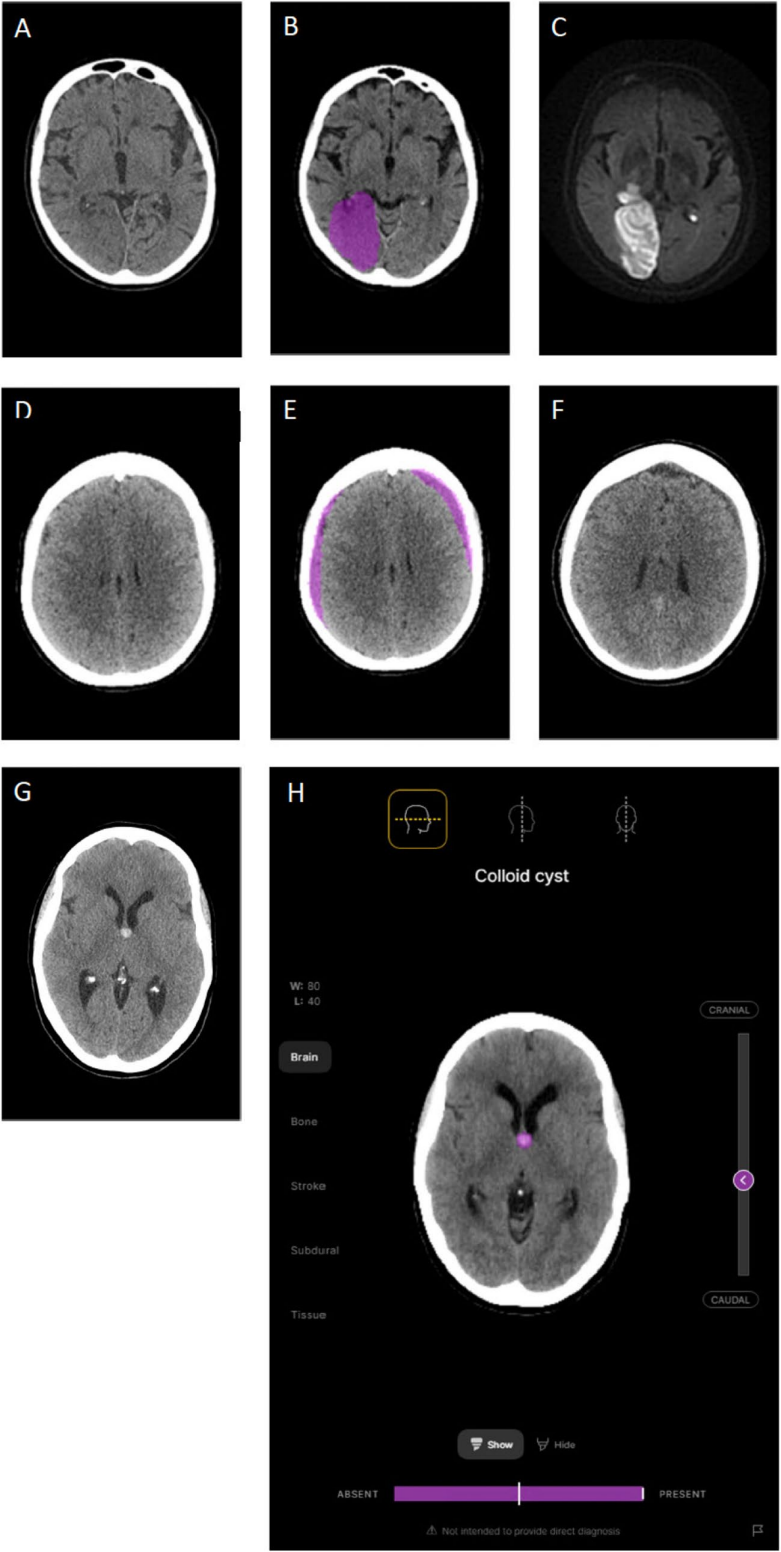
**A** Non-contrast CT brain study of a 79-year-old female who presented with acute stroke symptoms. Subtle hypodensity in the right occipital lobe was missed by 30 of the 32 readers in the unassisted arm of the study, but detected by 26 readers when using the deep learning tool as an assistant. **B** Output of the model. The model accurately localized the large area of infarction within the right occipital lobe (purple shading). **C** DWI image clearly showing the area of acute infarction in this patient. **D** An example of small bilateral isodense subacute subdural haematomas. **E** The haematomas were characterized by the model as subacute subdural haematomas and localized with purple shading. **F** A CT scan performed 7 days later. The haematoma is more conspicuous on the later scan as it evolves to become hypodense. **G** Non-contrast CT brain study demonstrating a colloid cyst. **H** The same colloid cyst case along with an example of the model’s segmentation and high confidence

**Fig. 5:**
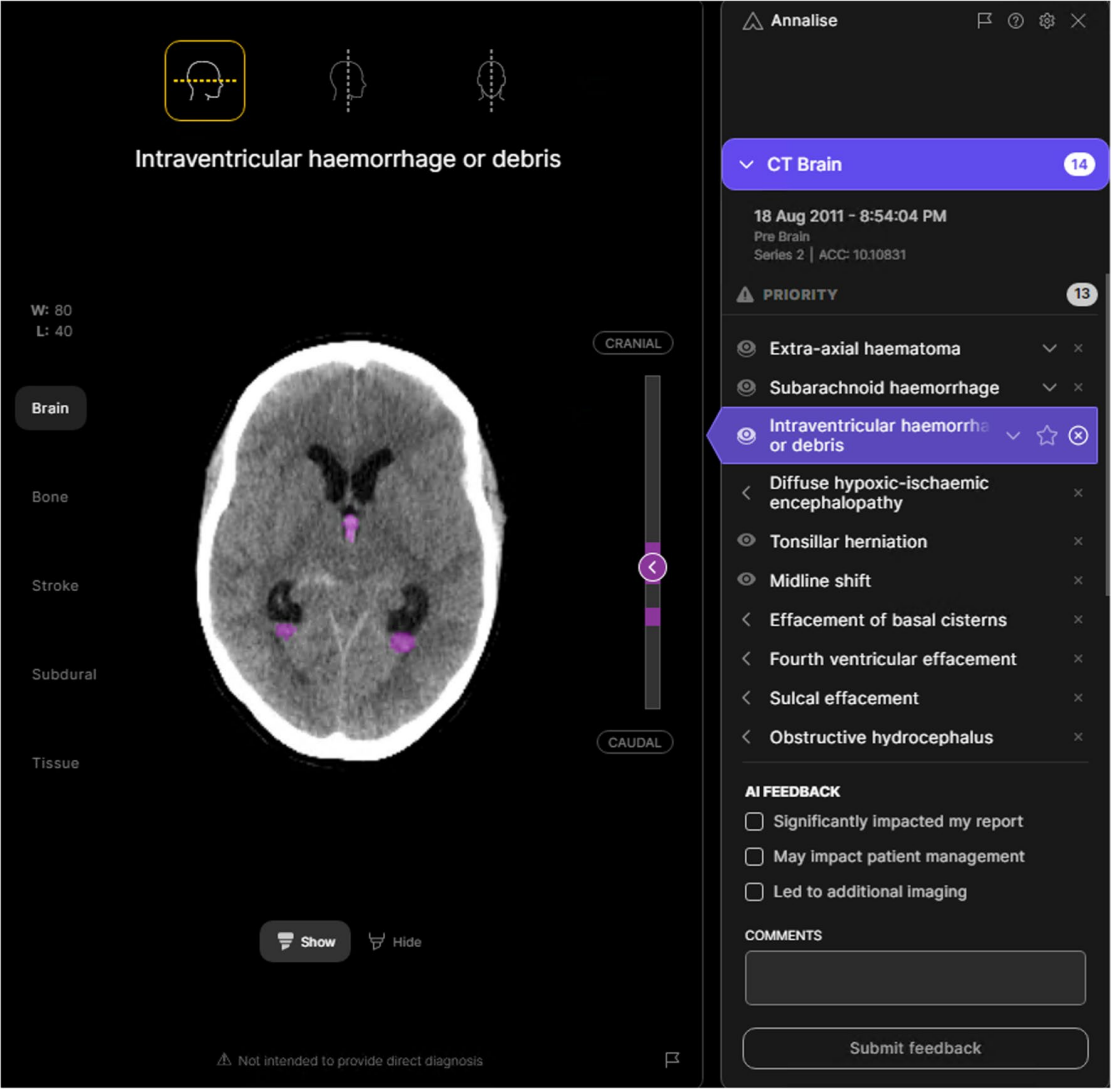
Non-contrast CT brain study demonstrating an intraventricular haemorrhage, along with an example of the decision support system’s user interface

**Fig. 6:**
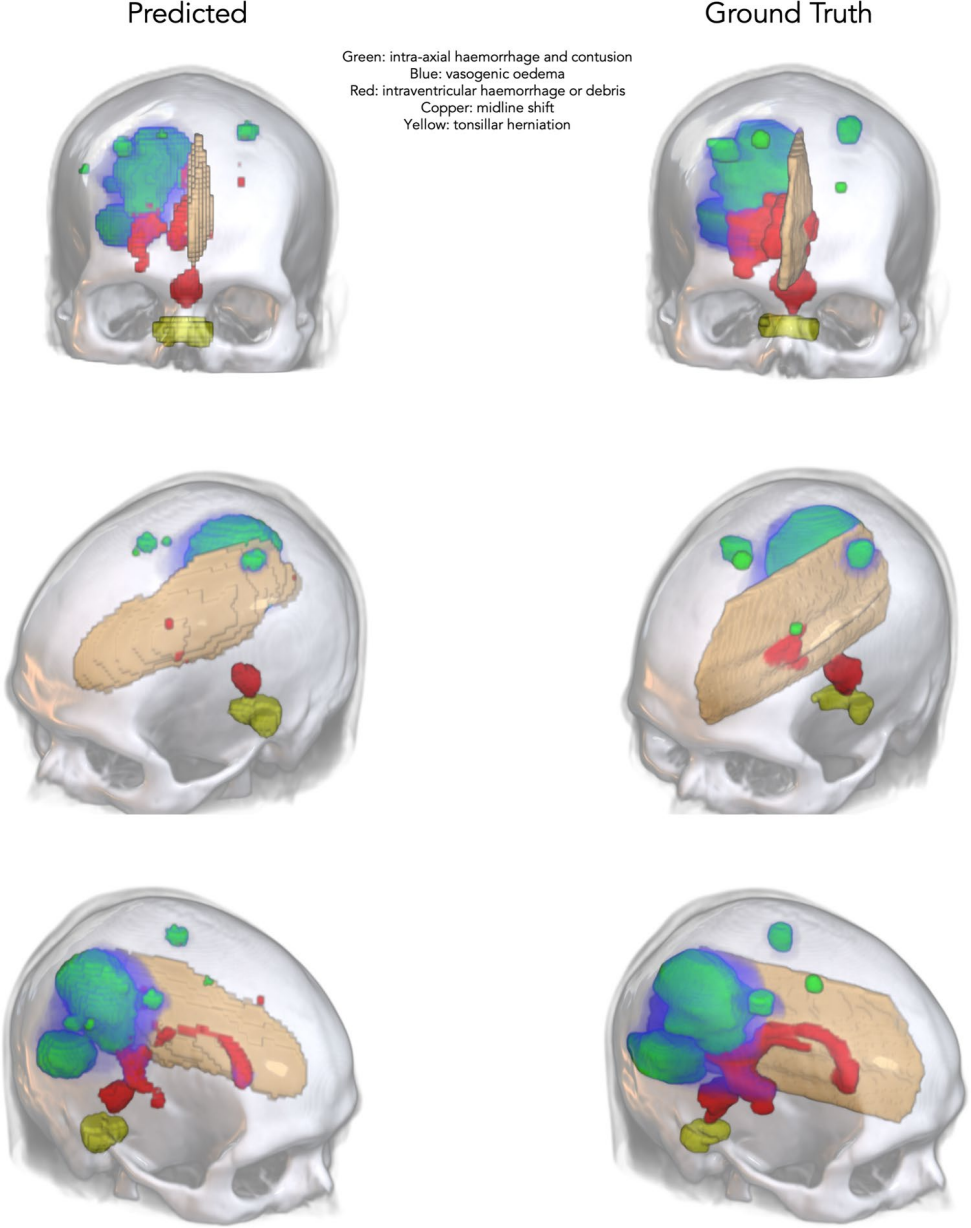
A three-dimensional (3D) visualisation of a single case containing multiple clinical findings demonstrating the 3D functionality of the model. The findings predicted by the model are presented alongside the ground-truth

Figure 3 demonstrates the effect of the model on radiologist recall and precision for all findings, averaged within the three groups based on the beta values chosen for each finding. Figure 4 presents an example case of acute cerebral infarction (A–C) with subtle NCCTB changes that were missed by most unassisted radiologists. This infarct was, however, identified by most radiologists when they used the deep learning tool. A subtle subacute subdural haematoma case (D–F) is also presented, along with model output and a scan from 7 days later. A colloid cyst case is presented, along with the model’s confidence (G–H). Figure 5 presents an intraventricular haemorrhage case. Figure 6 demonstrates the 3D functionality of the model, visualising a single case with multiple findings.

## Discussion

Since CT was invented there has been substantial improvement in spatial and contrast resolution, making it easier for clinicians to detect abnormalities. This study demonstrates a further advance in CT diagnostics through the use of comprehensive AI to aid a clinician’s detection of a range of abnormalities on NCCTBs. The developed model encompassed 144 clinical findings and was validated in a large-scale MRMC study. Reader performance when not assisted by the model, varied enormously depending on the subtlety and inherent subjectivity of the finding. The average AUC for unassisted readers across all findings was 0.68. The average AUC for the model alone was considerably higher at 0.93. When assisted by the model, radiologists significantly improved their performance across 91 clinical findings. One driver of high model accuracy was the large training dataset of 212,484 studies, each individually labelled for 192 findings by multiple radiologists.

Model benefits were most pronounced when aiding radiologists in the detection of subtle findings. The low unassisted radiologist AUC of 0.57 for “watershed infarct” indicated a performance that was little better than random guessing. Ground-truth labelling for acute infarcts was usually aided by diffusion-weighted MRI scans or follow-up CTs. Diffusion weighting is the most accurate method for detecting acute infarcts as it detects signal related to microscopic tissue changes. CT relies on macroscopic tissue changes to produce a change in density. However, as infarcts age, they become more visible, allowing for clearer detection on follow-up CT studies. Model performance for “watershed infarct,” with an AUC of 0.92 (0.88–0.94), indicated that although this finding proved difficult for radiologists to detect, subtle abnormalities were generally present on the CT scan that allowed detection by the model. The AUC for augmented readers was 0.68, while unaugmented readers demonstrated an average AUC of 0.57. The considerable improvement of the radiologists in detecting these infarcts when assisted by the model suggests that the findings on these studies were visible to the human eye even though they were often missed in the unassisted arm of the study. Further work is required to investigate the mechanisms driving the gap between augmented reader and model performance.

Interestingly, the model influenced the readers beyond just improving their accuracy. In radiology, there is often a trade-off between recall (i.e. sensitivity) and precision. The balance is usually struck with the level of precision being higher than the level of recall, typically because the majority of errors in radiology are errors of visual perception [27], which cause false negatives, and reduce recall. Visual search by clinicians favours some parts of the image over others. In contrast, CNNs tend to treat all parts of an image with the same level of scrutiny and can alert the radiologist to findings they would otherwise miss, raising their level of recall. We found that by changing the beta level of the model for the thresholds for different findings, which altered the balance of recall and precision of the model for those findings, we could alter the balance of recall and precision for the radiologists when assisted by the model. Beta levels were chosen based on the criticality of the finding. The logic was that for critical findings, the cost of a false positive is less than the cost of a false negative, so ideally, readers should favour recall over precision. For low criticality findings, precision would be favoured, and if the beta level was set to one, then recall and precision were weighted equally. As expected, readers favoured precision over recall in the unassisted arm of the study. In the assisted arm of the study, the ratio of precision to recall was determined largely by the beta levels chosen for the findings. For high criticality findings, recall was favoured over precision as desired, whereas for low criticality findings, precision remained favoured. Without losing precision, the recall of the high criticality findings markedly increased when assisted by the model. Thus, many of the largest gains in performance by the radiologists when assisted by the AI tool were for clinically critical findings.

The AUC of the model was greater for most findings than the mean AUC of assisted radiologists. This effect is well-recognized and is extensively described in the clinical decision support literature [10, 28]. Many findings in radiology are not entirely binary, and their presence or absence may be equivocal. The observation that radiologists did not always follow the predictions of the model most likely reflects the equivocal appearance of the findings on those cases. Many articles describe interobserver disagreement in radiology, which is particularly true of acute infarction [29, 30]. For findings such as extra-axial collection where appearance is less often equivocal, radiologist performance was similar to the model.

### Limitations and future directions

Despite the use of real-world data across multiple sites with varying demographics and different CT scanners, the study is retrospective in nature. A further limitation is that due to the comprehensive anonymisation of the study dataset, we were unable to perform consecutive case selection to better replicate real-world practice. Cases were instead randomly selected. A reduction in AUC of 0.05 was defined as clinically significant and only cerebellar agenesis reached this level of inferiority. The remaining 16 findings that were statistically inferior showed only minor reductions in AUC that were not clinically significant according to subspecialist neuroradiologists. The model’s benefits must be weighed against the possible general risks associated with the use of AI tools. Such risks (e.g. automation bias) could be realized if the user has little understanding of the tool or if the tool is used in an incorrect manner. It is ultimately the physician who must decide if a finding predicted by the model is truly present on the scan.

The main use for the CTB deep learning system will be to assist radiologists in their reporting of NCCTBs. The system could also be used for triage and in inpatient settings to assist clinicians in their decision-making at the point of care, particularly in low resource environments where expert radiologist advice may not be readily available.

## Conclusion

This study demonstrated that the use of a comprehensive AI-based software system in a controlled setting assisted radiologists in the detection of a range of abnormalities on non-contrast CT scans of the brain.

### Supplementary Information

Below is the link to the electronic supplementary material.

Supplementary file1 (DOCX 231 KB)

## Data Availability

The research team may make the model performance and radiologist performance datasets available to interested research partners with the goals of supporting the research community and making further collaborative contributions to the literature. Requests for access can be made through the annalise.ai website (https://annalise.ai/contact). The model is available as a commercial software product (https://annalise.ai/solutions/annalise-enterprise-ctb/). The free web-based demonstration can be accessed online.

## Abbreviations

3D: Three-dimensional
AI: Artificial intelligence
AUC: Area under the receiver operating characteristic curve
CI: Confidence interval
CNN: Convolutional neural networks
CT: Computed tomography
CTB: Computed tomography of the brain
DBS: Deep brain stimulation
DICOM: Digital Imaging and Communications in Medicine
DWI: Diffusion-weighted imaging
ED: Emergency department
FDA: US Food and Drug Administration
HREC: Human Research Ethics Committee
ICH: Intracranial haemorrhage
ID: Identification number
MCC: Matthews correlation coefficient
MRI: Magnetic resonance imaging
MRMC: Multi-reader multi-case
NCCTB: Non-contrast computed tomography of the brain
ROC: Receiver operating characteristic (curve)

## Code availability

The research team is unable to make the code underlying the system available as it is proprietary.

## Declarations

### Guarantor

The scientific guarantor of this publication is Prof. Peter Brotchie.

### Statistics and biometry

Prof. Graham Hepworth kindly provided statistical advice for this manuscript.

Numerous authors (PB, CT, JS, XH, AJ, BH, CJ, QB, NE, TG) have significant statistical expertise.

### Study subjects or cohorts overlap

No study subjects or cohorts have been previously reported.

### Methodology

• retrospective

• diagnostic study

• performed at one institution
