# Supplementary material for: Effects of a comprehensive brain computed tomography deep learning model on radiologist detection accuracy
Source: Eur Radiol. 2023 Aug 22;34(2):810–22. doi: 10.1007/s00330-023-10074-8 (PMC10853361; doi:10.1007/s00330-023-10074-8)
Supplement: Supplementary file 1 — Supplementary file1 (DOCX 231 KB) [file 330_2023_10074_MOESM1_ESM.docx]

**Effects of a comprehensive brain computed tomography deep learning model on radiologist detection accuracy**

**ELECTRONIC SUPPLEMENTARY MATERIAL**

Supplementary Tables

Supplementary Table 1: Ontology Tree identifying 192 child findings, the number of ground truth positive cases in the test dataset, and corresponding parent findings. A child finding is defined as a single radiological finding, corresponding to a single Leaf ID in the Ontology Tree. A parent finding is a conceptual group of multiple related child findings, which defines a logical relationship between a parent and its multiple children. Child findings with no corresponding parent finding are labelled with N/A (not applicable). * denotes that a finding was excluded from the final model. Findings that included localisation (laterality or segmentation) are emboldened. GT, ground truth.

| **Child Finding  (leaf identifier)** | **GT Positive Cases**  **(test dataset)** | **Parent Finding**  **(leaf identifier)** |
| --- | --- | --- |
| **abnormal_prominent_vessels** | 29 | N/A |
| acute_haemorrhagic_infarct | 24 | intraaxial_haemorrhage |
| **acute_infarct** | 248 | acute_infarction_in_the_brain |
| acute_infarct_petechial | 41 | acute_infarction_in_the_brain |
| acute_intraparenchymal_haemorrhage | 98 | intraaxial_haemorrhage |
| acute_lacunar_infarct | 85 | acute_infarction_in_the_brain |
| acute_on_chronic_subdural_haematoma | 49 | extra_axial_collection |
| acute_subdural_haematoma | 131 | extra_axial_collection |
| agenesis_corpus_callosum | 84 | N/A |
| aggressive_lesion_jaw* | 221 | N/A |
| aggressive_lytic_lesion_calvarial | 47 | aggressive_bone_lesion |
| **aggressive_meningeal_thickening** | 40 | extra_axial_mass |
| aggressive_mixed_lesion_calvarial | 40 | aggressive_bone_lesion |
| aggressive_sclerotic_lesion_calvarial* | 37 | aggressive_bone_lesion |
| aggressive_skin_lesion | 43 | skin_lesion |
| **aneurysm** | 43 | N/A |
| aneurysm_coils | 45 | N/A |
| atlanto-axial_subluxation* | 25 | N/A |
| atlanto-occipital_dissociation* | 3 | N/A |
| basal_cistern_effacement | 220 | N/A |
| basal_ganglia_calcification | 141 | N/A |
| basilar_thrombosis* | 15 | hyperdense_vessel |
| beam_hardening_artefact | 268 | N/A |
| benign_facial_bone_lesion* | 30 | benign_bone_lesion |
| benign_meningeal_thickening | 17 | N/A |
| benign_soft_tissue_mass_neck* | 36 | neck_mass_or_lesion |
| brainstem_atrophy* | 26 | N/A |
| brainstem_infarct | 32 | acute_infarction_in_the_brain |
| calvarial_thickening | 52 | N/A |
| cavum_septum_pellucidum | 107 | N/A |
| cerebellar_agenesis* | 36 | N/A |
| cerebellar_atrophy | 219 | N/A |
| cerebellar_infarct | 55 | acute_infarction_in_the_brain |
| cerebral_atrophy | 359 | N/A |
| cerebral_convexity_subarachnoid_haemorrhage | 137 | subarachnoid_haemorrhage |
| **chiari_1** | 41 | tonsillar_descent |
| chiari_2* | 45 | N/A |
| chronic_subdural_haematoma | 66 | extra_axial_collection |
| cochlear_implant | 6 | N/A |
| **colloid_cyst** | 39 | N/A |
| colpocephaly | 59 | N/A |
| communicating_hydrocephalus | 112 | N/A |
| confluent_white_matter_hypodensities | 6 | N/A |
| cortical_laminar_necrosis | 23 | N/A |
| cortical_leptomeningeal_calcification | 35 | N/A |
| craniotomy | 471 | N/A |
| craniotomy_collection | 146 | N/A |
| dbs_electrodes | 35 | N/A |
| deep_white_gray_matter_infarct | 51 | N/A |
| deep_white_matter_thinning* | 13 | N/A |
| **demyelination** | 9 | N/A |
| diffuse_axonal_injury* | 28 | N/A |
| **dilated_superior_opthalmic_vein** | 22 | N/A |
| dural_calcification | 129 | N/A |
| dural_venous_gas* | 16 | extra_axial_air |
| duret_haemorrhage* | 8 | N/A |
| empty_sella | 174 | N/A |
| encephalitis* | 32 | cerebral_oedema |
| encephalomalacia | 587 | N/A |
| enlarged_vestibular_acqueduct | 0 | N/A |
| entrapment_fourth_ventricle* | 16 | ventricular_entrapment |
| **entrapment_lateral_ventricle** | 39 | ventricular_entrapment |
| evd | 28 | N/A |
| **exophthalmos** | 47 | N/A |
| expanded_pituitary_fossa | 84 | N/A |
| extra_axial_collection_air | 138 | extra_axial_air |
| **extra_axial_collection_csf** | 203 | N/A |
| extra_axial_empyema* | 11 | extra_axial_collection |
| extra_axial_mass_calcific_density_non_aggressive | 91 | extra_axial_mass |
| extra_axial_mass_fat_density_non_aggressive | 21 | extra_axial_mass |
| **extra_axial_mass_soft_tissue_density_aggressive** | 52 | extra_axial_mass |
| extra_axial_mass_soft_tissue_density_non_aggressive | 39 | extra_axial_mass |
| extracranial_herniation | 35 | N/A |
| extradural_haematoma | 29 | extra_axial_collection |
| **extraocular_muscle_enlargement** | 47 | orbital_lesion |
| facial_bone_fracture | 119 | fracture |
| facial_bony_destruction | 59 | aggressive_bone_lesion |
| **fibrous_dysplasia** | 35 | benign_bone_lesion |
| **focal_calcification** | 90 | N/A |
| foreign_body_intracranial* | 26 | foreign_body |
| foreign_body_neck | 39 | foreign_body |
| foreign_body_orbit | 39 | foreign_body |
| foreign_body_scalp | 56 | foreign_body |
| fourth_ventricular_effacement | 75 | ventricular_effacement |
| fracture_C1-2* | 9 | fracture |
| fracture_calvarial | 45 | fracture |
| fracture_skull_base | 32 | fracture |
| fungal_sinusitis | 222 | N/A |
| **globe_shape_abnormality** | 69 | N/A |
| globus_pallidus_hypodensity* | 13 | N/A |
| haemorrhagic_contusion | 81 | intraaxial_haemorrhage |
| heterotopia* | 26 | N/A |
| **hyperdense_artery** | 24 | hyperdense_vessel |
| hyperostosis_frontalis | 181 | N/A |
| hypodense_basal_ganglia | 29 | N/A |
| hypopneumatised_mastoid | 146 | N/A |
| hypoxic_ischaemic_encephalopathy | 42 | N/A |
| icp | 0 | N/A |
| **insular_ribbon_sign** | 63 | N/A |
| **intra_ocular_silicone** | 41 | N/A |
| intraaxial_lesion_calcification | 24 | intraaxial_mass |
| intraaxial_lesion_complex_cyst | 32 | intraaxial_mass |
| intraaxial_lesion_CSF_cyst | 7 | intraaxial_mass |
| intraaxial_lesion_haemorrhage | 39 | intraaxial_mass |
| intraaxial_lesion_hyperdense | 44 | intraaxial_mass |
| intraaxial_lesion_hypodense | 25 | intraaxial_mass |
| intraaxial_lesion_isodense | 27 | intraaxial_mass |
| intraaxial_lesion_mixed | 49 | intraaxial_mass |
| intraventricular_debris* | 4 | intraventricular_haemorrhage_or  _debris |
| intraventricular_haemorrhage | 102 | intraventricular_haemorrhage_or  _debris |
| lacunar_infarct | 424 | N/A |
| **lateral_ventricular_effacement** | 335 | ventricular_effacement |
| longus_colli_calcification | 1 | N/A |
| lytic_lesion_skull_base | 83 | aggressive_bone_lesion |
| lytic_spine_lesion* | 40 | aggressive_bone_lesion |
| mastoid_opacity | 444 | N/A |
| **mastoidectomy** | 52 | N/A |
| meningioma_with_hyperostosis | 36 | extra_axial_mass |
| metallic_artefact | 546 | N/A |
| **midline_shift** | 214 | N/A |
| movement_artefact | 128 | N/A |
| mucocoele | 16 | N/A |
| mucosal_thickening | 1141 | N/A |
| neck_haematoma | 150 | N/A |
| non_aggressive_bone_lesion | 91 | benign_bone_lesion |
| non_aggressive_skin_lesion | 108 | skin_lesion |
| non_haemorrhagic_contusion* | 26 | N/A |
| obstructive_hydrocephalus | 96 | N/A |
| optic_neuritis* | 5 | N/A |
| **orbital_abscess*** | 8 | orbital_lesion |
| **orbital_fat_stranding** | 59 | N/A |
| orbital_mass | 41 | orbital_lesion |
| **orbital_prosthesis** | 52 | N/A |
| ossicular_chain_disruption* | 6 | N/A |
| **osteoma** | 95 | benign_bone_lesion |
| otic_capsule_trauma* | 4 | N/A |
| otosclerosis* | 9 | N/A |
| papilloedema* | 37 | N/A |
| **parotid_lesion** | 47 | neck_mass_or_lesion |
| periapical_abscess* | 57 | N/A |
| perimesencephalic_aneurysmal_subarachnoid_haemorrhage | 66 | subarachnoid_haemorrhage |
| peripheral_acute_infarct | 64 | acute_infarction_in_the_brain |
| perivascular_spaces | 338 | N/A |
| petrous_apex_lesion* | 40 | N/A |
| petrous_bone_fracture | 57 | fracture |
| **pineal_mass** | 22 | pineal_lesion |
| pseudo_sah* | 28 | N/A |
| **resection_cavity** | 85 | N/A |
| ruptured_dermoid* | 12 | extra_axial_mass |
| scalp_haematoma | 397 | N/A |
| schizencephaly* | 20 | N/A |
| scleral_thickening* | 41 | N/A |
| sclerotic_spine_lesion* | 39 | aggressive_bone_lesion |
| **sellar_haematoma** | 43 | sella_lesion_or_haematoma |
| sellar_lesion | 87 | sella_lesion_or_haematoma |
| **simple_pineal_cyst** | 58 | pineal_lesion |
| sino_nasal_surgery | 192 | N/A |
| sinus_fluid_level | 306 | N/A |
| sinus_lesion | 66 | N/A |
| skin_abscess* | 19 | skin_lesion |
| skull_vault_haemangioma | 64 | benign_bone_lesion |
| small_vessel_disease | 1081 | N/A |
| **soft_tissue_mass_neck** | 55 | neck_mass_or_lesion |
| stapes_implants* | 35 | N/A |
| striatocapsular_slit-like_chronic_hemorrhage | 38 | N/A |
| subacute_intraparenchymal_haemorrhage | 41 | intraaxial_haemorrhage |
| subacute_subdural_haematoma | 79 | extra_axial_collection |
| subcutaneous_emphysema | 167 | N/A |
| subependymal_calcification | 66 | N/A |
| sulcal_effacement | 679 | N/A |
| third_ventricular_effacement | 132 | ventricular_effacement |
| thornwaldt_cyst* | 23 | N/A |
| thyroid_eye_disease* | 27 | N/A |
| tmj_arthritis | 476 | N/A |
| **tmj_dislocation** | 46 | N/A |
| **tonsillar_herniation** | 38 | tonsillar_descent |
| transependymal_oedema | 24 | N/A |
| transphenoidal_surgery | 35 | N/A |
| **tympanic_cavity_erosion** | 21 | N/A |
| **tympanic_cavity_opacity** | 171 | N/A |
| **uncal_herniation** | 68 | N/A |
| upwards_transtentorial_herniation* | 28 | N/A |
| vascular_clips | 54 | N/A |
| vascular_stents* | 38 | N/A |
| **vasogenic_oedema** | 299 | cerebral_oedema |
| venous_infarct* | 8 | acute_infarction_in_the_brain |
| venous_thrombosis* | 24 | hyperdense_vessel |
| **ventricular_cyst** | 29 | N/A |
| **ventricular_mass** | 29 | intraaxial_mass |
| **vitreous_haemorrrhage** | 26 | N/A |
| vp_shunt | 193 | N/A |
| watershed_acute_infarct | 111 | acute_infarction_in_the_brain |

Supplementary Table 2: Ontology Tree identifying the 22 parent findings and number of ground truth positive cases in the test dataset, and corresponding parent findings. * denotes that a finding was excluded from the final model. Findings that included localisation (laterality or segmentation) are highlighted in bold.

| **Parent Finding** | **Identifier** | **GT+ve cases  (test set)** |
| --- | --- | --- |
| **Acute infarction in the brain** | **acute_infarction_in_the_brain*** | 363 |
| **Aggressive bone lesion** | **aggressive_bone_lesion*** | 221 |
| Benign bone lesion | benign_bone_lesion* | 297 |
| Cerebral oedema | cerebral _oedema* | 325 |
| **Extra axial air** | **extra_axial_air*** | 148 |
| Extra axial collection | extra_axial_collection* | 293 |
| Extra axial mass | extra_axial_mass* | 248 |
| **Foreign body** | **foreign_body*** | 121 |
| **Fracture** | **fracture*** | 196 |
| **Hyperdense vessel** | **hyperdense_vessel*** | 63 |
| Intraaxial haemorrhage | intraaxial_haemorrhage* | 220 |
| **Intraaxial mass** | **intraaxial_mass*** | 177 |
| **Intraventricular haemorrhage or debris** | **intraventricular_haemorrhage_or_debris*** | 105 |
| Neck mass or lesion | neck_mass_or_lesion* | 127 |
| Orbital lesion | orbital_lesion* | 93 |
| Pineal lesion | pineal_lesion* | 79 |
| Sellar lesion or haematoma | sellar_lesion_or_haematoma* | 100 |
| **Skin lesion** | **skin_lesion*** | 168 |
| **Subarachnoid haemorrhage** | **subarachnoid_haemorrhage** | 154 |
| Tonsillar descent | tonsillar_descent* | 79 |
| Ventricular effacement | ventricular_effacement* | 393 |
| Ventricular entrapment | ventricular_entrapment* | 54 |

Supplementary Table 3: Classification performance of the standalone model, unassisted radiologists and assisted radiologists reported as mean AUC and 95% Confidence Interval (95% CI), for the 189 ontology tree child findings evaluated. Findings labelled as “Not included” were not available as model predictions.

| **Finding (Leaf ID)** | **Standalone Model** | | **Radiologist Unassisted** | | **Radiologist Assisted** | | **Unassisted vs Assisted** | |
| --- | --- | --- | --- | --- | --- | --- | --- | --- |
|  | **Mean AUC** | **95% CI** | **Mean AUC** | **95% CI** | **Mean AUC** | **95% CI** | **Mean AUC Difference** | **95% CI** |
| abnormal_prominent_vessels | 0.77 | (0.68 - 0.85) | 0.55 | (0.52 - 0.59) | 0.55 | (0.52 - 0.58) | -0.01 | (-0.02, 0.01) |
| acute_haemorrhagic_infarct | 0.99 | (0.98 - 1) | 0.75 | (0.68 - 0.81) | 0.83 | (0.78 - 0.88) | 0.08 | (0.03, 0.14) |
| acute_infarct | 0.93 | (0.9 - 0.94) | 0.71 | (0.68 - 0.74) | 0.82 | (0.79 - 0.84) | 0.11 | (0.08, 0.14) |
| acute_infarct_petechial | 0.97 | (0.95 - 0.98) | 0.60 | (0.57 - 0.64) | 0.73 | (0.67 - 0.78) | 0.12 | (0.08, 0.17) |
| acute_intraparenchymal_haemorrhage | 0.98 | (0.98 - 0.99) | 0.84 | (0.8 - 0.87) | 0.91 | (0.88 - 0.94) | 0.07 | (0.03, 0.11) |
| acute_lacunar_infarct | 0.85 | (0.81 - 0.89) | 0.57 | (0.55 - 0.6) | 0.59 | (0.56 - 0.62) | 0.01 | (-0.01, 0.04) |
| acute_on_chronic_subdural_haematoma | 0.99 | (0.99 - 1) | 0.72 | (0.68 - 0.77) | 0.83 | (0.79 - 0.88) | 0.11 | (0.06, 0.16) |
| acute_subdural_haematoma | 0.97 | (0.96 - 0.98) | 0.77 | (0.74 - 0.8) | 0.89 | (0.86 - 0.91) | 0.12 | (0.08, 0.15) |
| agenesis_corpus_callosum | 0.99 | (0.98 - 1) | 0.78 | (0.74 - 0.82) | 0.82 | (0.78 - 0.86) | 0.04 | (0, 0.08) |
| aggressive_lesion_jaw | Not included | | 0.59 | (0.55 - 0.63) | 0.58 | (0.54 - 0.61) | -0.01 | (-0.03, 0.01) |
| aggressive_lytic_lesion_calvarial | 0.95 | (0.91 - 0.98) | 0.78 | (0.73 - 0.82) | 0.82 | (0.77 - 0.87) | 0.04 | (0.02, 0.07) |
| aggressive_meningeal_thickening | 0.91 | (0.87 - 0.94) | 0.56 | (0.53 - 0.59) | 0.59 | (0.55 - 0.63) | 0.03 | (0, 0.06) |
| aggressive_mixed_lesion_calvarial | 0.86 | (0.8 - 0.91) | 0.64 | (0.61 - 0.68) | 0.59 | (0.56 - 0.62) | -0.05 | (-0.08, -0.03) |
| aggressive_sclerotic_lesion_calvarial | Not included | | 0.66 | (0.61 - 0.7) | 0.66 | (0.61 - 0.71) | 0.00 | (-0.03, 0.03) |
| aggressive_skin_lesion | 0.95 | (0.91 - 0.97) | 0.70 | (0.65 - 0.74) | 0.76 | (0.71 - 0.81) | 0.06 | (0.02, 0.1) |
| aneurysm | 0.82 | (0.74 - 0.88) | 0.59 | (0.56 - 0.63) | 0.66 | (0.61 - 0.72) | 0.07 | (0.03, 0.1) |
| aneurysm_coils | 1.00 | (1 - 1) | 0.95 | (0.93 - 0.98) | 0.97 | (0.94 - 0.99) | 0.01 | (0, 0.03) |
| atlanto_axial_subluxation | Not included | | 0.54 | (0.52 - 0.57) | 0.52 | (0.5 - 0.54) | -0.02 | (-0.04, 0) |
| atlanto_occipital_dissociation | Not included | | 0.56 | (0.48 - 0.64) | 0.56 | (0.49 - 0.62) | -0.01 | (-0.08, 0.07) |
| basal_cistern_effacement | 0.97 | (0.96 - 0.98) | 0.61 | (0.58 - 0.63) | 0.78 | (0.75 - 0.81) | 0.17 | (0.13, 0.21) |
| basal_ganglia_calcification | 0.96 | (0.95 - 0.98) | 0.75 | (0.71 - 0.79) | 0.81 | (0.77 - 0.85) | 0.06 | (0.03, 0.09) |
| basilar_thrombosis | Not included | | 0.55 | (0.51 - 0.59) | 0.53 | (0.5 - 0.55) | -0.02 | (-0.05, 0.01) |
| beam_hardening_artefact | 0.79 | (0.76 - 0.82) | 0.53 | (0.51 - 0.54) | 0.56 | (0.54 - 0.58) | 0.03 | (0.01, 0.04) |
| benign_facial_bone_lesion | Not included | | 0.52 | (0.51 - 0.53) | 0.52 | (0.51 - 0.53) | 0.00 | (-0.01, 0.01) |
| benign_meningeal_thickening | 0.90 | (0.83 - 0.97) | 0.51 | (0.5 - 0.52) | 0.51 | (0.5 - 0.52) | 0.00 | (-0.02, 0.01) |
| benign_soft_tissue_mass_neck | Not included | | 0.55 | (0.52 - 0.57) | 0.56 | (0.53 - 0.58) | 0.01 | (-0.01, 0.03) |
| tympanic_cavity_erosion | Not included | | 0.58 | (0.55 - 0.62) | 0.55 | (0.53 - 0.58) | -0.03 | (-0.05, -0.01) |
| brainstem_infarct | 0.80 | (0.73 - 0.87) | 0.53 | (0.51 - 0.55) | 0.54 | (0.52 - 0.57) | 0.01 | (-0.01, 0.03) |
| calvarial_thickening | 0.97 | (0.95 - 0.99) | 0.69 | (0.64 - 0.73) | 0.67 | (0.63 - 0.72) | -0.01 | (-0.05, 0.02) |
| cavum_septum_pellucidum | 0.75 | (0.69 - 0.8) | 0.65 | (0.61 - 0.69) | 0.62 | (0.59 - 0.66) | -0.03 | (-0.04, -0.01) |
| cerebellar_agenesis | 0.92 | (0.9 - 0.94) | 0.76 | (0.71 - 0.81) | 0.65 | (0.6 - 0.7) | -0.11 | (-0.15, -0.06) |
| cerebellar_atrophy | 0.91 | (0.87 - 0.95) | 0.61 | (0.59 - 0.64) | 0.67 | (0.65 - 0.7) | 0.06 | (0.04, 0.08) |
| cerebellar_infarct | Not included | | 0.66 | (0.61 - 0.7) | 0.73 | (0.67 - 0.78) | 0.07 | (0.03, 0.11) |
| cerebral_atrophy | 0.88 | (0.86 - 0.9) | 0.61 | (0.58 - 0.63) | 0.66 | (0.63 - 0.68) | 0.05 | (0.03, 0.07) |
| cerebral_convexity_subarachnoid_haemorrhage | 0.96 | (0.94 - 0.98) | 0.76 | (0.72 - 0.79) | 0.89 | (0.86 - 0.91) | 0.13 | (0.09, 0.17) |
| chiari_1 | 0.98 | (0.97 - 0.99) | 0.68 | (0.63 - 0.73) | 0.74 | (0.68 - 0.8) | 0.06 | (0, 0.12) |
| chiari_2 | Not included | | 0.73 | (0.68 - 0.79) | 0.70 | (0.64 - 0.77) | -0.03 | (-0.07, 0.01) |
| chronic_subdural_haematoma | 0.97 | (0.95 - 0.99) | 0.69 | (0.65 - 0.73) | 0.83 | (0.79 - 0.86) | 0.14 | (0.09, 0.19) |
| cochlear_implant | 1.00 | (1 - 1) | 0.92 | (0.88 - 0.96) | 0.93 | (0.86 - 1.01) | 0.02 | (-0.06, 0.09) |
| colloid_cyst | 1.00 | (0.99 - 1) | 0.86 | (0.81 - 0.9) | 0.96 | (0.92 - 0.99) | 0.10 | (0.05, 0.15) |
| colpocephaly | 0.99 | (0.98 - 0.99) | 0.77 | (0.73 - 0.82) | 0.78 | (0.73 - 0.82) | 0.00 | (-0.03, 0.04) |
| communicating_hydrocephalus | 0.97 | (0.97 - 0.98) | 0.72 | (0.69 - 0.76) | 0.77 | (0.73 - 0.8) | 0.04 | (0.01, 0.08) |
| confluent_white_matter_hypodensities | 0.94 | (0.87 - 1) | 0.61 | (0.52 - 0.7) | 0.58 | (0.51 - 0.66) | -0.03 | (-0.07, 0.02) |
| cortical_laminar_necrosis | 0.95 | (0.93 - 0.97) | 0.60 | (0.55 - 0.65) | 0.58 | (0.54 - 0.62) | -0.02 | (-0.04, 0.01) |
| cortical_leptomeningeal_calcification | 0.95 | (0.88 - 0.98) | 0.68 | (0.62 - 0.73) | 0.67 | (0.61 - 0.72) | -0.01 | (-0.04, 0.02) |
| craniotomy | 0.99 | (0.99 - 0.99) | 0.92 | (0.9 - 0.93) | 0.95 | (0.93 - 0.96) | 0.03 | (0.02, 0.04) |
| craniotomy_collection | 0.99 | (0.98 - 0.99) | 0.79 | (0.76 - 0.82) | 0.88 | (0.86 - 0.9) | 0.09 | (0.05, 0.12) |
| dbs_electrodes | 1.00 | (1 - 1) | 0.97 | (0.95 - 0.99) | 0.99 | (0.99 - 1) | 0.02 | (0, 0.04) |
| deep_white_gray_matter_infarct | 0.96 | (0.92 - 0.98) | 0.72 | (0.67 - 0.76) | 0.75 | (0.69 - 0.8) | 0.03 | (0, 0.06) |
| deep_white_matter_thinning | Not included | | 0.58 | (0.54 - 0.61) | 0.56 | (0.52 - 0.6) | -0.02 | (-0.05, 0.01) |
| demyelination | 0.84 | (0.7 - 0.95) | 0.57 | (0.5 - 0.65) | 0.54 | (0.49 - 0.58) | -0.04 | (-0.08, 0) |
| diffuse_axonal_injury | Not included | | 0.73 | (0.67 - 0.79) | 0.68 | (0.62 - 0.74) | -0.05 | (-0.09, -0.02) |
| dilated_superior_opthalmic_vein | 0.78 | (0.68 - 0.87) | 0.61 | (0.56 - 0.67) | 0.63 | (0.57 - 0.69) | 0.02 | (-0.01, 0.05) |
| dural_calcification | 0.94 | (0.92 - 0.96) | 0.59 | (0.57 - 0.62) | 0.70 | (0.66 - 0.73) | 0.10 | (0.06, 0.14) |
| dural_venous_gas | Not included | | 0.65 | (0.58 - 0.71) | 0.62 | (0.57 - 0.67) | -0.03 | (-0.06, 0) |
| duret_haemorrhage | Not included | | 0.68 | (0.62 - 0.74) | 0.63 | (0.57 - 0.69) | -0.05 | (-0.1, 0) |
| empty_sella | 0.96 | (0.94 - 0.97) | 0.73 | (0.7 - 0.76) | 0.84 | (0.81 - 0.87) | 0.11 | (0.07, 0.15) |
| encephalitis | Not included | | 0.53 | (0.5 - 0.56) | 0.53 | (0.51 - 0.55) | -0.01 | (-0.02, 0.01) |
| encephalomalacia | 0.96 | (0.95 - 0.96) | 0.77 | (0.75 - 0.79) | 0.85 | (0.84 - 0.87) | 0.08 | (0.06, 0.1) |
| entrapment_fourth_ventricle | Not included | | 0.58 | (0.53 - 0.62) | 0.55 | (0.52 - 0.58) | -0.03 | (-0.06, 0.01) |
| entrapment_lateral_ventricle | 0.98 | (0.97 - 0.99) | 0.60 | (0.55 - 0.64) | 0.64 | (0.59 - 0.69) | 0.05 | (0, 0.09) |
| evd | 0.99 | (0.99 - 1) | 0.78 | (0.73 - 0.83) | 0.82 | (0.77 - 0.87) | 0.04 | (-0.02, 0.1) |
| exophthalmos | 0.98 | (0.97 - 0.99) | 0.64 | (0.6 - 0.68) | 0.64 | (0.6 - 0.69) | 0.00 | (-0.03, 0.03) |
| expanded_pituitary_fossa | 0.98 | (0.97 - 0.99) | 0.74 | (0.7 - 0.78) | 0.83 | (0.79 - 0.86) | 0.08 | (0.04, 0.12) |
| extra_axial_collection_air | 0.94 | (0.91 - 0.96) | 0.83 | (0.8 - 0.86) | 0.88 | (0.86 - 0.91) | 0.06 | (0.03, 0.08) |
| extra_axial_collection_csf | 0.96 | (0.94 - 0.97) | 0.69 | (0.66 - 0.72) | 0.79 | (0.76 - 0.82) | 0.10 | (0.06, 0.13) |
| extra_axial_empyema | Not included | | 0.61 | (0.53 - 0.69) | 0.59 | (0.53 - 0.65) | -0.02 | (-0.05, 0.02) |
| extra_axial_mass_calcific_density_non_aggressive | 0.94 | (0.92 - 0.96) | 0.75 | (0.71 - 0.79) | 0.81 | (0.77 - 0.85) | 0.06 | (0.03, 0.09) |
| extra_axial_mass_fat_density_non_aggressive | 0.74 | (0.63 - 0.85) | 0.72 | (0.64 - 0.79) | 0.70 | (0.62 - 0.78) | -0.02 | (-0.05, 0.02) |
| extra_axial_mass_soft_tissue_density_aggressive | 0.96 | (0.92 - 0.98) | 0.66 | (0.62 - 0.7) | 0.75 | (0.7 - 0.8) | 0.09 | (0.05, 0.13) |
| extra_axial_mass_soft_tissue_density_non_aggressive | 0.84 | (0.77 - 0.89) | 0.58 | (0.55 - 0.62) | 0.64 | (0.59 - 0.68) | 0.05 | (0.01, 0.09) |
| extracranial_herniation | 0.96 | (0.93 - 0.98) | 0.76 | (0.7 - 0.81) | 0.75 | (0.68 - 0.81) | -0.01 | (-0.04, 0.02) |
| extradural_haematoma | 0.94 | (0.89 - 0.97) | 0.72 | (0.66 - 0.78) | 0.67 | (0.61 - 0.73) | -0.05 | (-0.09, -0.02) |
| extraocular_muscle_enlargement | 0.93 | (0.88 - 0.97) | 0.70 | (0.66 - 0.74) | 0.78 | (0.72 - 0.83) | 0.08 | (0.03, 0.12) |
| facial_bone_fracture | 0.90 | (0.87 - 0.94) | 0.79 | (0.76 - 0.83) | 0.83 | (0.8 - 0.86) | 0.04 | (0.01, 0.06) |
| facial_bony_destruction | 0.94 | (0.9 - 0.97) | 0.69 | (0.64 - 0.73) | 0.77 | (0.73 - 0.81) | 0.08 | (0.04, 0.12) |
| fibrous_dysplasia | 0.79 | (0.7 - 0.88) | 0.67 | (0.62 - 0.71) | 0.65 | (0.6 - 0.7) | -0.02 | (-0.05, 0.01) |
| focal_calcification | 0.92 | (0.88 - 0.95) | 0.65 | (0.62 - 0.68) | 0.80 | (0.77 - 0.84) | 0.15 | (0.1, 0.2) |
| foreign_body_intracranial | Not included | | 0.78 | (0.72 - 0.84) | 0.79 | (0.73 - 0.85) | 0.01 | (-0.02, 0.05) |
| foreign_body_neck | 0.84 | (0.75 - 0.92) | 0.61 | (0.57 - 0.65) | 0.64 | (0.59 - 0.69) | 0.03 | (-0.01, 0.07) |
| foreign_body_orbit | 0.93 | (0.89 - 0.96) | 0.72 | (0.67 - 0.76) | 0.76 | (0.7 - 0.81) | 0.04 | (0, 0.08) |
| foreign_body_scalp | 0.84 | (0.77 - 0.9) | 0.62 | (0.58 - 0.65) | 0.67 | (0.63 - 0.72) | 0.06 | (0.02, 0.09) |
| fourth_ventricular_effacement | 0.98 | (0.97 - 0.99) | 0.69 | (0.64 - 0.73) | 0.85 | (0.81 - 0.89) | 0.17 | (0.12, 0.21) |
| fracture_c1_2 | Not included | | 0.62 | (0.54 - 0.69) | 0.57 | (0.52 - 0.62) | -0.05 | (-0.09, -0.01) |
| fracture_calvarial | 0.94 | (0.9 - 0.98) | 0.73 | (0.69 - 0.78) | 0.82 | (0.76 - 0.87) | 0.08 | (0.04, 0.13) |
| fracture_skull_base | 0.92 | (0.85 - 0.97) | 0.62 | (0.58 - 0.66) | 0.68 | (0.63 - 0.74) | 0.06 | (0.01, 0.11) |
| fungal_sinusitis | 0.97 | (0.96 - 0.98) | 0.70 | (0.67 - 0.74) | 0.81 | (0.78 - 0.84) | 0.11 | (0.07, 0.14) |
| globe_shape_abnormality | 0.93 | (0.89 - 0.96) | 0.69 | (0.65 - 0.73) | 0.80 | (0.76 - 0.84) | 0.11 | (0.06, 0.16) |
| globus_pallidus_hypodensity | Not included | | 0.73 | (0.65 - 0.8) | 0.69 | (0.62 - 0.77) | -0.03 | (-0.08, 0.02) |
| haemorrhagic_contusion | 0.98 | (0.96 - 0.99) | 0.74 | (0.7 - 0.78) | 0.87 | (0.84 - 0.9) | 0.13 | (0.08, 0.17) |
| heterotopia | Not included | | 0.64 | (0.58 - 0.69) | 0.63 | (0.57 - 0.69) | -0.01 | (-0.03, 0.02) |
| hyperdense_artery | 0.96 | (0.94 - 0.98) | 0.57 | (0.54 - 0.61) | 0.72 | (0.65 - 0.79) | 0.15 | (0.08, 0.21) |
| hyperostosis_frontalis | 0.99 | (0.98 - 0.99) | 0.67 | (0.63 - 0.7) | 0.80 | (0.76 - 0.83) | 0.13 | (0.08, 0.18) |
| hypodense_basal_ganglia | 0.96 | (0.91 - 0.98) | 0.57 | (0.53 - 0.61) | 0.73 | (0.67 - 0.8) | 0.16 | (0.1, 0.22) |
| hypopneumatised_mastoid | 0.97 | (0.95 - 0.98) | 0.73 | (0.69 - 0.77) | 0.76 | (0.73 - 0.8) | 0.03 | (0, 0.07) |
| hypoxic_ischaemic_encephalopathy | 0.98 | (0.97 - 0.99) | 0.82 | (0.76 - 0.87) | 0.81 | (0.75 - 0.87) | -0.01 | (-0.03, 0.02) |
| insular_ribbon_sign | 0.96 | (0.95 - 0.98) | 0.55 | (0.53 - 0.57) | 0.72 | (0.68 - 0.77) | 0.17 | (0.11, 0.23) |
| intra_ocular_silicone | 1.00 | (1 - 1) | 0.89 | (0.85 - 0.93) | 0.91 | (0.88 - 0.95) | 0.03 | (-0.01, 0.06) |
| intraaxial_lesion_calcification | 0.96 | (0.94 - 0.98) | 0.70 | (0.64 - 0.76) | 0.77 | (0.7 - 0.84) | 0.07 | (0.02, 0.13) |
| intraaxial_lesion_complex_cyst | 0.95 | (0.9 - 0.98) | 0.71 | (0.65 - 0.77) | 0.78 | (0.72 - 0.84) | 0.07 | (0.03, 0.12) |
| intraaxial_lesion_csf_cyst | 0.82 | (0.62 - 0.99) | 0.66 | (0.54 - 0.78) | 0.60 | (0.49 - 0.72) | -0.06 | (-0.11, 0) |
| intraaxial_lesion_haemorrhage | 0.97 | (0.95 - 0.98) | 0.70 | (0.66 - 0.75) | 0.81 | (0.76 - 0.86) | 0.10 | (0.06, 0.15) |
| intraaxial_lesion_hyperdense | 0.96 | (0.94 - 0.97) | 0.69 | (0.65 - 0.73) | 0.74 | (0.7 - 0.79) | 0.05 | (0.02, 0.09) |
| intraaxial_lesion_hypodense | 0.90 | (0.82 - 0.96) | 0.57 | (0.54 - 0.6) | 0.67 | (0.62 - 0.73) | 0.10 | (0.05, 0.15) |
| intraaxial_lesion_isodense | 0.91 | (0.86 - 0.96) | 0.58 | (0.54 - 0.61) | 0.66 | (0.61 - 0.7) | 0.08 | (0.03, 0.13) |
| intraaxial_lesion_mixed | 0.97 | (0.96 - 0.98) | 0.67 | (0.63 - 0.71) | 0.79 | (0.75 - 0.84) | 0.12 | (0.07, 0.17) |
| intraventricular_debris | Not included | | 0.50 | (0.49 - 0.51) | 0.50 | (0.5 - 0.5) | 0.00 | (-0.01, 0) |
| intraventricular_haemorrhage | 0.99 | (0.98 - 1) | 0.81 | (0.77 - 0.85) | 0.92 | (0.89 - 0.94) | 0.11 | (0.07, 0.15) |
| lacunar_infarct | 0.92 | (0.9 - 0.93) | 0.69 | (0.66 - 0.71) | 0.77 | (0.75 - 0.79) | 0.08 | (0.05, 0.11) |
| lateral_ventricular_effacement | 0.98 | (0.97 - 0.98) | 0.75 | (0.72 - 0.77) | 0.88 | (0.86 - 0.9) | 0.14 | (0.1, 0.17) |
| lytic_lesion_skull_base | 0.90 | (0.85 - 0.93) | 0.68 | (0.64 - 0.71) | 0.74 | (0.7 - 0.78) | 0.06 | (0.03, 0.09) |
| lytic_spine_lesion | Not included | | 0.62 | (0.58 - 0.66) | 0.59 | (0.56 - 0.63) | -0.03 | (-0.06, 0) |
| mastoid_opacity | 0.91 | (0.89 - 0.92) | 0.73 | (0.71 - 0.76) | 0.78 | (0.76 - 0.8) | 0.04 | (0.02, 0.07) |
| mastoidectomy | 0.99 | (0.97 - 1) | 0.80 | (0.76 - 0.85) | 0.94 | (0.91 - 0.96) | 0.13 | (0.09, 0.18) |
| meningioma_with_hyperostosis | 0.96 | (0.93 - 0.98) | 0.61 | (0.57 - 0.65) | 0.60 | (0.56 - 0.65) | -0.01 | (-0.03, 0.02) |
| metallic_artefact | 0.97 | (0.96 - 0.98) | 0.77 | (0.74 - 0.8) | 0.83 | (0.81 - 0.86) | 0.07 | (0.04, 0.09) |
| midline_shift | 0.98 | (0.97 - 0.99) | 0.75 | (0.72 - 0.78) | 0.91 | (0.89 - 0.93) | 0.16 | (0.12, 0.2) |
| movement_artefact | 0.91 | (0.87 - 0.94) | 0.69 | (0.66 - 0.73) | 0.71 | (0.68 - 0.75) | 0.02 | (-0.01, 0.05) |
| mucocoele | 0.97 | (0.94 - 0.99) | 0.67 | (0.62 - 0.73) | 0.66 | (0.6 - 0.72) | -0.01 | (-0.05, 0.03) |
| mucosal_thickening | 0.90 | (0.89 - 0.91) | 0.79 | (0.77 - 0.8) | 0.78 | (0.77 - 0.8) | 0.00 | (-0.02, 0.01) |
| neck_haematoma | 0.96 | (0.94 - 0.97) | 0.75 | (0.72 - 0.78) | 0.85 | (0.82 - 0.88) | 0.10 | (0.06, 0.13) |
| non_aggressive_bone_lesion | 0.74 | (0.68 - 0.79) | 0.60 | (0.57 - 0.63) | 0.57 | (0.55 - 0.59) | -0.03 | (-0.05, -0.01) |
| non_aggressive_skin_lesion | 0.86 | (0.81 - 0.91) | 0.69 | (0.65 - 0.72) | 0.70 | (0.66 - 0.73) | 0.01 | (-0.01, 0.03) |
| non_haemorrhagic_contusion | Not included | | 0.62 | (0.57 - 0.67) | 0.59 | (0.55 - 0.63) | -0.03 | (-0.06, 0) |
| obstructive_hydrocephalus | 0.98 | (0.97 - 0.99) | 0.63 | (0.6 - 0.66) | 0.80 | (0.76 - 0.84) | 0.17 | (0.13, 0.21) |
| optic_neuritis | Not included | | 0.55 | (0.5 - 0.6) | 0.55 | (0.51 - 0.58) | -0.01 | (-0.04, 0.03) |
| orbital_abscess | Not included | | 0.59 | (0.53 - 0.65) | 0.57 | (0.5 - 0.63) | -0.02 | (-0.04, 0) |
| orbital_fat_stranding | 0.95 | (0.92 - 0.97) | 0.66 | (0.63 - 0.7) | 0.75 | (0.7 - 0.8) | 0.09 | (0.04, 0.14) |
| orbital_mass | 0.88 | (0.82 - 0.93) | 0.68 | (0.63 - 0.73) | 0.71 | (0.65 - 0.77) | 0.03 | (0, 0.06) |
| orbital_prosthesis | 1.00 | (0.99 - 1) | 0.92 | (0.89 - 0.95) | 0.96 | (0.93 - 0.98) | 0.03 | (0.01, 0.06) |
| ossicular_chain_disruption | Not included | | 0.57 | (0.52 - 0.62) | 0.54 | (0.5 - 0.57) | -0.03 | (-0.06, 0) |
| osteoma | 0.83 | (0.77 - 0.89) | 0.64 | (0.61 - 0.67) | 0.69 | (0.65 - 0.73) | 0.05 | (0.02, 0.08) |
| otic_capsule_trauma | Not included | | 0.60 | (0.52 - 0.67) | 0.58 | (0.53 - 0.62) | -0.02 | (-0.09, 0.06) |
| otosclerosis | Not included | | 0.57 | (0.5 - 0.64) | 0.53 | (0.5 - 0.55) | -0.05 | (-0.1, 0.01) |
| papilloedema | Not included | | 0.57 | (0.54 - 0.61) | 0.57 | (0.53 - 0.61) | 0.00 | (-0.03, 0.02) |
| parotid_lesion | 0.80 | (0.72 - 0.87) | 0.64 | (0.6 - 0.68) | 0.63 | (0.59 - 0.67) | -0.01 | (-0.04, 0.02) |
| periapical_abscess | Not included | | 0.59 | (0.56 - 0.62) | 0.57 | (0.54 - 0.6) | -0.02 | (-0.04, 0) |
| perimesencephalic_aneurysmal_subarachnoid_haemorrhage | 0.99 | (0.98 - 1) | 0.71 | (0.67 - 0.76) | 0.83 | (0.79 - 0.88) | 0.12 | (0.07, 0.16) |
| peripheral_acute_infarct | 0.89 | (0.85 - 0.92) | 0.51 | (0.5 - 0.52) | 0.51 | (0.5 - 0.52) | 0.00 | (-0.01, 0.01) |
| perivascular_spaces | 0.88 | (0.86 - 0.9) | 0.59 | (0.57 - 0.61) | 0.60 | (0.58 - 0.63) | 0.01 | (-0.01, 0.03) |
| petrous_apex_lesion | Not included | | 0.58 | (0.54 - 0.61) | 0.57 | (0.54 - 0.6) | -0.01 | (-0.03, 0.01) |
| petrous_bone_fracture | 0.96 | (0.93 - 0.98) | 0.76 | (0.71 - 0.8) | 0.84 | (0.79 - 0.88) | 0.08 | (0.03, 0.13) |
| pineal_mass | 0.81 | (0.7 - 0.9) | 0.63 | (0.56 - 0.69) | 0.65 | (0.58 - 0.73) | 0.03 | (-0.03, 0.09) |
| pseudo_sah | Not included | | 0.75 | (0.68 - 0.81) | 0.78 | (0.72 - 0.85) | 0.04 | (0, 0.07) |
| resection_cavity | 0.98 | (0.97 - 0.99) | 0.69 | (0.65 - 0.72) | 0.73 | (0.69 - 0.77) | 0.04 | (0.01, 0.08) |
| ruptured_dermoid | Not included | | 0.57 | (0.53 - 0.6) | 0.56 | (0.51 - 0.6) | -0.01 | (-0.03, 0.01) |
| scalp_haematoma | 0.96 | (0.94 - 0.97) | 0.72 | (0.7 - 0.75) | 0.82 | (0.8 - 0.84) | 0.10 | (0.07, 0.13) |
| schizencephaly | Not included | | 0.77 | (0.67 - 0.86) | 0.76 | (0.67 - 0.85) | -0.01 | (-0.03, 0.02) |
| scleral_thickening | Not included | | 0.55 | (0.52 - 0.57) | 0.54 | (0.52 - 0.56) | -0.01 | (-0.02, 0) |
| sclerotic_spine_lesion | Not included | | 0.63 | (0.59 - 0.67) | 0.62 | (0.57 - 0.67) | -0.01 | (-0.04, 0.02) |
| sellar_haematoma | 0.88 | (0.79 - 0.95) | 0.74 | (0.69 - 0.78) | 0.76 | (0.71 - 0.81) | 0.02 | (-0.01, 0.05) |
| sellar_lesion | 0.96 | (0.93 - 0.98) | *0.78* | (0.74 - 0.82) | *0.85* | (0.81 - 0.88) | 0.06 | (0.03, 0.1) |
| simple_pineal_cyst | 0.95 | (0.91 - 0.98) | 0.62 | (0.57 - 0.66) | 0.72 | (0.67 - 0.77) | 0.10 | (0.06, 0.15) |
| sino_nasal_surgery | 0.87 | (0.85 - 0.9) | 0.63 | (0.61 - 0.66) | 0.74 | (0.71 - 0.77) | 0.10 | (0.07, 0.13) |
| sinus_fluid_level | 0.94 | (0.93 - 0.95) | 0.77 | (0.74 - 0.79) | 0.85 | (0.83 - 0.88) | 0.09 | (0.06, 0.11) |
| sinus_lesion | 0.92 | (0.88 - 0.95) | 0.67 | (0.64 - 0.71) | 0.73 | (0.69 - 0.78) | 0.06 | (0.02, 0.1) |
| skin_abscess | Not included | | 0.67 | (0.61 - 0.73) | 0.61 | (0.56 - 0.66) | -0.06 | (-0.1, -0.01) |
| skull_vault_haemangioma | 0.80 | (0.75 - 0.84) | 0.61 | (0.58 - 0.65) | 0.58 | (0.55 - 0.61) | -0.03 | (-0.06, 0) |
| small_vessel_disease | 0.94 | (0.93 - 0.95) | 0.73 | (0.7 - 0.75) | 0.83 | (0.82 - 0.85) | 0.11 | (0.08, 0.13) |
| soft_tissue_mass_neck | 0.87 | (0.81 - 0.93) | 0.66 | (0.62 - 0.7) | 0.71 | (0.66 - 0.76) | 0.06 | (0.02, 0.1) |
| stapes_implants | Not included | | 0.68 | (0.62 - 0.73) | 0.69 | (0.64 - 0.74) | 0.01 | (-0.02, 0.05) |
| striatocapsular_slit_like_chronic_hemorrhage | 0.96 | (0.93 - 0.98) | 0.60 | (0.56 - 0.64) | 0.58 | (0.54 - 0.61) | -0.03 | (-0.06, 0) |
| subacute_intraparenchymal_haemorrhage | 0.93 | (0.9 - 0.96) | 0.62 | (0.58 - 0.66) | 0.62 | (0.58 - 0.66) | 0.00 | (-0.02, 0.03) |
| subacute_subdural_haematoma | 0.98 | (0.97 - 0.99) | 0.75 | (0.71 - 0.79) | 0.82 | (0.78 - 0.86) | 0.07 | (0.04, 0.1) |
| subcutaneous_emphysema | 0.93 | (0.92 - 0.95) | 0.66 | (0.63 - 0.7) | 0.81 | (0.78 - 0.84) | 0.14 | (0.1, 0.19) |
| subependymal_calcification | 0.89 | (0.84 - 0.93) | 0.70 | (0.66 - 0.74) | 0.69 | (0.65 - 0.74) | -0.01 | (-0.03, 0.01) |
| sulcal_effacement | 0.96 | (0.96 - 0.97) | 0.69 | (0.66 - 0.71) | 0.88 | (0.86 - 0.89) | 0.19 | (0.16, 0.22) |
| third_ventricular_effacement | 0.98 | (0.98 - 0.99) | 0.71 | (0.67 - 0.74) | 0.83 | (0.8 - 0.86) | 0.12 | (0.09, 0.16) |
| thornwaldt_cyst | Not included | | 0.52 | (0.51 - 0.54) | 0.52 | (0.51 - 0.54) | 0.00 | (-0.01, 0.01) |
| thyroid_eye_disease | Not included | | 0.74 | (0.68 - 0.81) | 0.72 | (0.65 - 0.78) | -0.03 | (-0.06, 0.01) |
| tmj_arthritis | 0.85 | (0.83 - 0.87) | 0.71 | (0.68 - 0.74) | 0.71 | (0.68 - 0.74) | 0.00 | (-0.03, 0.02) |
| tmj_dislocation | 0.96 | (0.93 - 0.98) | 0.73 | (0.69 - 0.77) | 0.69 | (0.65 - 0.74) | -0.04 | (-0.08, 0) |
| tonsillar_herniation | 0.99 | (0.98 - 0.99) | 0.65 | (0.6 - 0.7) | 0.83 | (0.78 - 0.89) | 0.19 | (0.12, 0.25) |
| transependymal_oedema | 0.98 | (0.96 - 0.99) | 0.65 | (0.6 - 0.7) | 0.74 | (0.68 - 0.81) | 0.09 | (0.04, 0.15) |
| transphenoidal_surgery | 0.96 | (0.93 - 0.99) | 0.78 | (0.73 - 0.84) | 0.81 | (0.75 - 0.87) | 0.03 | (-0.01, 0.07) |
| tympanic_cavity_erosion | 0.99 | (0.98 - 0.99) | 0.65 | (0.6 - 0.69) | 0.62 | (0.57 - 0.66) | -0.03 | (-0.06, 0) |
| tympanic_cavity_opacity | 0.99 | (0.97 - 1) | 0.76 | (0.73 - 0.8) | 0.90 | (0.88 - 0.92) | 0.14 | (0.1, 0.18) |
| uncal_herniation | 0.99 | (0.99 - 1) | 0.70 | (0.65 - 0.74) | 0.89 | (0.85 - 0.92) | 0.19 | (0.13, 0.26) |
| upwards_transtentorial_herniation | Not included | | 0.60 | (0.55 - 0.64) | 0.60 | (0.55 - 0.65) | 0.00 | (-0.02, 0.03) |
| vascular_clips | 0.98 | (0.97 - 0.99) | 0.80 | (0.76 - 0.85) | 0.87 | (0.82 - 0.91) | 0.06 | (0.03, 0.09) |
| vascular_stents | Not included | | 0.73 | (0.68 - 0.79) | 0.72 | (0.66 - 0.77) | -0.02 | (-0.05, 0.01) |
| vasogenic_oedema | 0.98 | (0.97 - 0.98) | 0.81 | (0.79 - 0.84) | 0.91 | (0.89 - 0.92) | 0.09 | (0.07, 0.12) |
| venous_infarct | Not included | | 0.61 | (0.55 - 0.66) | 0.59 | (0.54 - 0.64) | -0.02 | (-0.07, 0.03) |
| venous_thrombosis | Not included | | 0.61 | (0.56 - 0.67) | 0.60 | (0.55 - 0.65) | -0.02 | (-0.05, 0.01) |
| ventricular_cyst | 0.79 | (0.69 - 0.87) | 0.62 | (0.57 - 0.67) | 0.64 | (0.59 - 0.68) | 0.02 | (-0.02, 0.05) |
| ventricular_mass | 0.85 | (0.76 - 0.92) | 0.65 | (0.59 - 0.71) | 0.62 | (0.57 - 0.67) | -0.03 | (-0.05, 0) |
| vitreous_haemorrrhage | 0.97 | (0.94 - 0.99) | 0.85 | (0.8 - 0.9) | 0.87 | (0.82 - 0.93) | 0.02 | (-0.01, 0.06) |
| vp_shunt | 1.00 | (0.99 - 1) | 0.96 | (0.95 - 0.97) | 0.97 | (0.95 - 0.98) | 0.00 | (-0.01, 0.01) |
| watershed_acute_infarct | 0.92 | (0.88 - 0.94) | 0.57 | (0.55 - 0.6) | 0.68 | (0.64 - 0.72) | 0.11 | (0.07, 0.15) |

Supplementary Table 4: Classification performance of the standalone model, unassisted radiologists and assisted radiologists reported as mean AUC and 95% Confidence Interval (95% CI) for the 22 parent findings.

|  | **Standalone  Model** | | | **Radiologist Unassisted** | | | **Radiologist Assisted** | | | | |
| --- | --- | --- | --- | --- | --- | --- | --- | --- | --- | --- | --- |
| **Finding (Leaf ID)** | **Mean AUC** | | **95% CI** | **Mean AUC** | | **95% CI** | | **Mean AUC** | **95% CI** |  |  |
| acute_infarction_in_the_brain | 0.91 | (0.9 - 0.93) | | 0.72 | (0.7 - 0.74) | | | 0.80 | (0.77 - 0.82) | | |
| aggressive_bone_lesion | 0.86 | (0.83 - 0.89) | | 0.76 | (0.74 - 0.79) | | | 0.77 | (0.74 - 0.79) | | |
| benign_bone_lesion | 0.70 | (0.67 - 0.74) | | 0.64 | (0.62 - 0.66) | | | 0.63 | (0.61 - 0.65) | | |
| cerebral_oedema | 0.95 | (0.94 - 0.97) | | 0.79 | (0.77 - 0.81) | | | 0.88 | (0.86 - 0.89) | | |
| extra_axial_air | 0.93 | (0.9 - 0.95) | | 0.83 | (0.79 - 0.85) | | | 0.87 | (0.85 - 0.9) | | |
| extra_axial_collection | 0.94 | (0.92 - 0.95) | | 0.84 | (0.81 - 0.86) | | | 0.92 | (0.9 - 0.93) | | |
| extra_axial_mass | 0.86 | (0.83 - 0.89) | | 0.72 | (0.69 - 0.74) | | | 0.77 | (0.74 - 0.8) | | |
| foreign_body | 0.84 | (0.79 - 0.88) | | 0.69 | (0.65 - 0.71) | | | 0.72 | (0.68 - 0.75) | | |
| fracture | 0.91 | (0.88 - 0.93) | | 0.78 | (0.75 - 0.81) | | | 0.84 | (0.81 - 0.86) | | |
| hyperdense_vessel | 0.82 | (0.77 - 0.87) | | 0.59 | (0.55 - 0.61) | | | 0.63 | (0.59 - 0.66) | | |
| intraaxial_haemorrhage | 0.91 | (0.89 - 0.93) | | 0.84 | (0.81 - 0.86) | | | 0.90 | (0.88 - 0.92) | | |
| intraaxial_mass | 0.97 | (0.96 - 0.98) | | 0.77 | (0.74 - 0.79) | | | 0.81 | (0.78 - 0.83) | | |
| intraventricular_haemorrhage_or_debris | 0.98 | (0.97 - 0.99) | | 0.81 | (0.77 - 0.84) | | | 0.91 | (0.88 - 0.93) | | |
| neck_mass_or_lesion | 0.78 | (0.73 - 0.83) | | 0.64 | (0.61 - 0.66) | | | 0.65 | (0.62 - 0.68) | | |
| orbital_lesion | 0.92 | (0.89 - 0.95) | | 0.72 | (0.68 - 0.75) | | | 0.76 | (0.71 - 0.79) | | |
| pineal_lesion | 0.88 | (0.83 - 0.92) | | 0.66 | (0.62 - 0.7) | | | 0.74 | (0.7 - 0.78) | | |
| sellar_lesion_or_haematoma | 0.93 | (0.9 - 0.96) | | 0.79 | (0.76 - 0.82) | | | 0.85 | (0.81 - 0.88) | | |
| skin_lesion | 0.85 | (0.82 - 0.89) | | 0.70 | (0.67 - 0.72) | | | 0.71 | (0.68 - 0.74) | | |
| subarachnoid_haemorrhage | 0.97 | (0.95 - 0.98) | | 0.78 | (0.74 - 0.8) | | | 0.89 | (0.86 - 0.91) | | |
| tonsillar_descent | 0.98 | (0.97 - 0.99) | | 0.67 | (0.63 - 0.7) | | | 0.79 | (0.74 - 0.82) | | |
| ventricular_effacement | 0.97 | (0.97 - 0.98) | | 0.74 | (0.72 - 0.76) | | | 0.88 | (0.86 - 0.9) | | |
| ventricular_entrapment | 0.93 | (0.89 - 0.96) | | 0.59 | (0.56 - 0.62) | | | 0.62 | (0.58 - 0.65) | | |

Supplementary Table 5: Child ontology tree findings (n=48) not included in the CTB model and reason for exclusion

| **Finding (Leaf ID)** | **Reason for exclusion as a Model Finding** |
| --- | --- |
| aggressive_lesion_jaw | Val AUC = 0.67 |
| aggressive_sclerotic_lesion_calvarial | Could not achieve precision of 0.2, Val AUC = 0.78 |
| atlanto-axial_subluxation | 14 Training cases, could not achieve precision of 0.2, Val AUC = 0.71 |
| atlanto-occipital_dissociation | 2 Training cases, 3 test cases, could not achieve precision of 0.2, Val AUC = 0.64 |
| basilar_thrombosis | 15 Test cases, could not achieve precision of 0.2 |
| benign_facial_bone_lesion | Could not achieve precision of 0.2, Val AUC = 0.66 |
| benign_soft_tissue_mass_neck | Could not achieve precision of 0.2, Val AUC = 0.59 |
| brainstem_atrophy | Excluded due to poor performance on clinical review |
| cerebellar_agenesis | Excluded due to poor performance on clinical review |
| chiari_2 | 17 Training cases, could not achieve precision of 0.2 |
| deep_white_matter_thinning | 10 Training cases, 13 Test cases, could not achieve precision of 0.2 |
| diffuse_axonal_injury | 34 Training cases |
| dural_venous_gas | 19 Training cases, 16 Test cases, could not achieve precision of 0.2, Val AUC = 0.60 |
| duret_haemorrhage | 15 Training cases, 8 Test cases |
| encephalitis | Could not achieve precision of 0.2, Val AUC = 0.78 |
| enlarged_vestibular_acqueduct | 11 Training cases, 0 Test cases, could not achieve precision of 0.2, Val AUC = 0.35 |
| entrapment_fourth_ventricle | 7 Training cases, 16 Test cases, could not achieve precision of 0.2, Val AUC = 0.33 |
| extra_axial_empyema | 10 Training cases, 11 Test cases, could not achieve precision of 0.2, Val AUC = 0.67 |
| foreign_body_intracranial | 15 Training cases, could not achieve precision of 0.2, Val AUC = 0.63 |
| fracture_C1-2 | 9 Test cases |
| globus_pallidus_hypodensity | 9 Training cases, 13 Test cases, could not achieve precision of 0.2, Val AUC = 0.65 |
| heterotopia | 17 Training cases, could not achieve precision of 0.2, Val AUC = 0.63 |
| icp | 7 Training cases, 0 Test cases, could not achieve precision of 0.2, Val AUC = 0.62 |
| intraventricular_debris | 3 Training cases, 4 Test cases, could not achieve precision of 0.2, Val AUC = 0.796 |
| longus_colli_calcification | 0 Training cases, 1 Test cases, could not achieve precision of 0.2, Cannot calculate validation AUC |
| lytic_spine_lesion | Could not achieve precision of 0.2, Val AUC 0.66 |
| non_haemorrhagic_contusion | Could not achieve precision of 0.2 |
| optic_neuritis | 18 Training cases, 5 Test cases, could not achieve precision of 0.2, Val AUC = 0.69 |
| orbital_abscess | 2 Training cases, 8 Test cases, could not achieve precision of 0.2, Val AUC = 0.73 |
| ossicular_chain_disruption | 9 Training cases, 6 Test cases, could not achieve precision of 0.2, Val AUC = 0.49 |
| otic_capsule_trauma | 9 Training cases, 4 Test cases, could not achieve precision of 0.2, Val AUC = 0.77 |
| otosclerosis | 11 Training cases, 9 Test cases, could not achieve precision of 0.2, Val AUC = 0.48 |
| papilloedema | 41 Training cases, could not achieve precision of 0.2, Val AUC = 0.59 |
| periapical_abscess | Could not achieve precision of 0.2, Val AUC = 0.78 |
| petrous_apex_lesion | Val AUC = 0.76 |
| pseudo_sah | 19 Training cases |
| ruptured_dermoid | 29 Training cases, 12 Test cases, could not achieve precision of 0.2, Val AUC = 0.53 |
| schizencephaly | 15 Training cases, could not achieve precision of 0.2, Val AUC = 0.60 |
| scleral_thickening | 35 Training cases, could not achieve precision of 0.2, Val AUC = 0.74 |
| sclerotic_spine_lesion | Could not achieve precision of 0.2, Val AUC = 0.72 |
| skin_abscess | 28 Training cases, 19 Test cases, could not achieve precision of 0.2, Val AUC = 0.66 |
| stapes_implants | Could not achieve precision of 0.2, Val AUC = 0.57 |
| thornwaldt_cyst | 22 Training cases, could not achieve precision of 0.2, Val AUC = 0.47 |
| thyroid_eye_disease | Excluded due to poor performance on clinical review |
| upwards_transtentorial_herniation | 20 Training cases, could not achieve precision of 0.2 |
| vascular_stents | Could not achieve precision of 0.2 |
| venous_infarct | 7 Training cases, 8 Test cases, could not achieve precision of 0.2, Val AUC = 0.55 |
| venous_thrombosis | Could not achieve precision of 0.2, Val AUC = 0.70 |
| **Total Findings not included** | **48 child findings** |

Supplementary Table 6: Performance of the unassisted and assisted radiologists reported as mean sensitivity, specificity and positive predictive value for the 144 ontology tree child findings selected for inclusion in the AI model. Mean delta represents the change in AUC between the unassisted and assisted radiologists. GT, ground truth.

|  |  | | **Sensitivity** | | | **Specificity** | | | | **Positive Predictive Value** | | | |
| --- | --- | --- | --- | --- | --- | --- | --- | --- | --- | --- | --- | --- | --- |
|  | GT +ve cases | Mean standalone model | | Mean unassisted | Mean assisted | | Mean standalone model | Mean unassisted | Mean assisted | | Mean standalone model | Mean unassisted | Mean assisted |
| abnormal_prominent_vessels | 29 | 0.0 | | 0.11 | 0.10 | | 1.00 | 1.00 | 1.00 | | 0.00 | 0.37 | 0.43 |
| acute_haemorrhagic_infarct | 24 | 0.79 | | 0.50 | 0.67 | | 0.99 | 0.99 | 0.99 | | 0.37 | 0.37 | 0.38 |
| acute_infarct | 248 | 0.81 | | 0.45 | 0.67 | | 0.92 | 0.96 | 0.95 | | 0.49 | 0.54 | 0.56 |
| acute_infarct_petechial | 41 | 0.59 | | 0.21 | 0.46 | | 0.98 | 0.99 | 0.99 | | 0.35 | 0.39 | 0.41 |
| acute_intraparenchymal_haemorrhage | 98 | 0.94 | | 0.68 | 0.84 | | 0.93 | 0.93 | 0.94 | | 0.32 | 0.55 | 0.61 |
| acute_lacunar_infarct | 85 | 0.26 | | 0.17 | 0.19 | | 0.98 | 0.98 | 0.96 | | 0.26 | 0.60 | 0.49 |
| acute_on_chronic_subdural_haematoma | 49 | 0.98 | | 0.45 | 0.68 | | 0.96 | 0.97 | 0.98 | | 0.28 | 0.22 | 0.29 |
| acute_subdural_haematoma | 131 | 0.93 | | 0.56 | 0.80 | | 0.92 | 0.99 | 0.98 | | 0.36 | 0.61 | 0.47 |
| agenesis_corpus_callosum | 84 | 0.57 | | 0.56 | 0.64 | | 1.00 | 0.98 | 0.96 | | 0.92 | 0.62 | 0.51 |
| aggressive_lytic_lesion_calvarial | 47 | 0.58 | | 0.56 | 0.65 | | 0.98 | 0.99 | 1.00 | | 0.37 | 0.77 | 0.86 |
| aggressive_meningeal_thickening | 40 | 0.03 | | 0.13 | 0.19 | | 1.00 | 0.97 | 0.98 | | 0.26 | 0.64 | 0.70 |
| aggressive_mixed_lesion_calvarial | 40 | 0.00 | | 0.29 | 0.18 | | 1.00 | 1.00 | 1.00 | | N/A | 0.38 | 0.46 |
| aggressive_skin_lesion | 43 | 0.42 | | 0.40 | 0.52 | | 0.99 | 0.99 | 0.99 | | 0.43 | 0.49 | 0.49 |
| aneurysm | 43 | 0.41 | | 0.19 | 0.34 | | 0.97 | 1.00 | 1.00 | | 0.16 | 0.53 | 0.45 |
| aneurysm_coils | 45 | 0.78 | | 0.91 | 0.93 | | 1.00 | 0.99 | 1.00 | | 0.97 | 0.36 | 0.45 |
| basal_cistern_effacement | 220 | 0.82 | | 0.22 | 0.58 | | 0.96 | 0.99 | 0.99 | | 0.66 | 0.34 | 0.39 |
| basal_ganglia_calcification | 141 | 0.64 | | 0.52 | 0.64 | | 0.99 | 0.99 | 0.99 | | 0.69 | 0.57 | 0.52 |
| beam_hardening_artefact | 268 | 0.08 | | 0.07 | 0.12 | | 1.00 | 0.99 | 0.98 | | 0.62 | 0.47 | 0.28 |
| benign_meningeal_thickening | 17 | 0.00 | | 0.03 | 0.02 | | 1.00 | 1.00 | 1.00 | | N/A | 0.84 | 0.92 |
| brainstem_infarct | 32 | 0.19 | | 0.06 | 0.09 | | 0.98 | 1.00 | 1.00 | | 0.11 | 0.57 | 0.58 |
| calvarial_thickening | 52 | 0.20 | | 0.38 | 0.35 | | 1.00 | 1.00 | 1.00 | | 0.91 | 0.25 | 0.42 |
| cavum_septum_pellucidum | 107 | 0.12 | | 0.32 | 0.25 | | 1.00 | 1.00 | 0.98 | | 1.00 | 0.83 | 0.70 |
| cerebellar_atrophy | 219 | 0.365 | | 0.27 | 0.38 | | 0.99 | 0.98 | 0.98 | | 0.72 | 0.66 | 0.65 |
| cerebellar_infarct | 55 | 0.53 | | 0.32 | 0.46 | | 0.99 | 1.00 | 1.00 | | 0.49 | 0.42 | 0.50 |
| cerebral_atrophy | 359 | 0.43 | | 0.27 | 0.36 | | 0.96 | 0.99 | 0.99 | | 0.59 | 0.54 | 0.65 |
| cerebral_convexity_subarachnoid_haemorrhage | 137 | 0.93 | | 0.53 | 0.80 | | 0.87 | 0.97 | 0.98 | | 0.27 | 0.59 | 0.70 |
| chiari_1 | 41 | 0.29 | | 0.37 | 0.49 | | 1.00 | 1.00 | 1.00 | | 0.44 | 0.15 | 0.21 |
| chronic_subdural_haematoma | 66 | 0.86 | | 0.39 | 0.67 | | 0.97 | 1.00 | 1.00 | | 0.38 | 0.07 | 0.09 |
| cochlear_implant | 6 | 0.75 | | 0.83 | 0.87 | | 1.00 | 1.00 | 1.00 | | 1.00 | 0.30 | 0.27 |
| colloid_cyst | 39 | 0.90 | | 0.72 | 0.91 | | 1.00 | 0.99 | 1.00 | | 0.83 | 0.23 | 0.18 |
| colpocephaly | 59 | 0.33 | | 0.55 | 0.56 | | 1.00 | 1.00 | 0.99 | | 0.83 | 0.15 | 0.17 |
| communicating_hydrocephalus | 112 | 0.61 | | 0.46 | 0.55 | | 0.98 | 0.99 | 1.00 | | 0.62 | 0.51 | 0.75 |
| confluent_white_matter_hypodensities | 6 | 0.00 | | 0.23 | 0.17 | | 1.00 | 0.98 | 1.00 | | N/A | 0.52 | 0.75 |
| cortical_laminar_necrosis | 23 | 0.00 | | 0.21 | 0.17 | | 1.00 | 0.99 | 1.00 | | N/A | 0.59 | 0.72 |
| cortical_leptomeningeal_calcification | 35 | 0.09 | | 0.36 | 0.34 | | 1.00 | 0.96 | 0.97 | | 0.50 | 0.45 | 0.58 |
| craniotomy | 471 | 0.89 | | 0.84 | 0.90 | | 0.99 | 0.99 | 0.99 | | 0.93 | 0.56 | 0.61 |
| craniotomy_collection | 146 | 0.77 | | 0.59 | 0.77 | | 0.99 | 0.94 | 0.95 | | 0.76 | 0.49 | 0.57 |
| dbs_electrodes | 35 | 0.97 | | 0.94 | 0.98 | | 1.00 | 0.99 | 0.95 | | 1.00 | 0.69 | 0.49 |
| deep_white_gray_matter_infarct | 51 | 0.47 | | 0.45 | 0.50 | | 1.00 | 0.95 | 0.91 | | 0.80 | 0.64 | 0.58 |
| demyelination | 9 | 0.00 | | 0.16 | 0.08 | | 1.00 | 0.99 | 0.99 | | N/A | 0.36 | 0.37 |
| dilated_superior_opthalmic_vein | 22 | 0.00 | | 0.23 | 0.27 | | 1.00 | 1.00 | 1.00 | | N/A | 0.77 | 0.81 |
| dural_calcification | 129 | 0.44 | | 0.20 | 0.40 | | 0.98 | 0.99 | 0.98 | | 0.55 | 0.45 | 0.42 |
| empty_sella | 174 | 0.72 | | 0.53 | 0.73 | | 0.96 | 1.00 | 1.00 | | 0.52 | 0.89 | 0.94 |
| encephalomalacia | 587 | 0.77 | | 0.58 | 0.73 | | 0.96 | 1.00 | 0.99 | | 0.84 | 0.84 | 0.77 |
| entrapment_lateral_ventricle | 39 | 0.44 | | 0.20 | 0.29 | | 1.00 | 0.99 | 1.00 | | 0.81 | 0.72 | 0.77 |
| evd | 28 | 0.50 | | 0.56 | 0.64 | | 0.99 | 0.98 | 0.98 | | 0.47 | 0.54 | 0.59 |
| exophthalmos | 47 | 0.11 | | 0.29 | 0.29 | | 1.00 | 0.98 | 1.00 | | 0.63 | 0.09 | 0.21 |
| expanded_pituitary_fossa | 84 | 0.57 | | 0.50 | 0.67 | | 0.99 | 0.99 | 1.00 | | 0.68 | 0.27 | 0.28 |
| extra_axial_collection_air | 138 | 0.75 | | 0.66 | 0.77 | | 0.96 | 0.99 | 0.99 | | 0.49 | 0.51 | 0.54 |
| extra_axial_collection_csf | 203 | 0.56 | | 0.40 | 0.59 | | 0.99 | 0.99 | 0.99 | | 0.85 | 0.94 | 0.95 |
| extra_axial_mass_calcific_density_non_aggressive | 91 | 0.60 | | 0.51 | 0.64 | | 0.98 | 0.99 | 0.99 | | 0.49 | 0.79 | 0.77 |
| extra_axial_mass_fat_density_non_aggressive | 21 | 0.00 | | 0.43 | 0.40 | | 1.00 | 1.00 | 1.00 | | N/A | 0.98 | 0.99 |
| extra_axial_mass_soft_tissue_density_aggressive | 52 | 0.47 | | 0.33 | 0.50 | | 0.99 | 0.99 | 0.99 | | 0.48 | 0.52 | 0.66 |
| extra_axial_mass_soft_tissue_density_ non_aggressive | 39 | 0.34 | | 0.17 | 0.28 | | 0.98 | 1.00 | 1.00 | | 0.19 | 0.30 | 0.20 |
| extracranial_herniation | 35 | 0.21 | | 0.52 | 0.50 | | 1.00 | 0.98 | 1.00 | | 1.00 | 0.09 | 0.17 |
| extradural_haematoma | 29 | 0.07 | | 0.44 | 0.34 | | 1.00 | 0.99 | 1.00 | | 0.40 | 0.56 | 0.64 |
| extraocular_muscle_enlargement | 47 | 0.51 | | 0.40 | 0.56 | | 0.99 | 0.99 | 0.99 | | 0.41 | 0.50 | 0.57 |
| facial_bone_fracture | 119 | 0.59 | | 0.59 | 0.67 | | 0.99 | 0.99 | 0.99 | | 0.69 | 0.59 | 0.61 |
| facial_bony_destruction | 59 | 0.45 | | 0.38 | 0.55 | | 0.99 | 1.00 | 1.00 | | 0.57 | 0.52 | 0.53 |
| fibrous_dysplasia | 35 | 0.11 | | 0.34 | 0.30 | | 1.00 | 1.00 | 1.00 | | 0.80 | 0.43 | 0.46 |
| focal_calcification | 90 | 0.78 | | 0.31 | 0.63 | | 0.94 | 0.92 | 0.93 | | 0.29 | 0.37 | 0.45 |
| foreign_body_neck | 39 | 0.13 | | 0.22 | 0.28 | | 1.00 | 0.98 | 0.99 | | 0.39 | 0.21 | 0.34 |
| foreign_body_orbit | 39 | 0.42 | | 0.43 | 0.52 | | 0.99 | 0.96 | 0.96 | | 0.37 | 0.81 | 0.82 |
| foreign_body_scalp | 56 | 0.23 | | 0.24 | 0.35 | | 0.99 | 1.00 | 1.00 | | 0.45 | 0.37 | 0.36 |
| fourth_ventricular_effacement | 75 | 0.81 | | 0.37 | 0.72 | | 0.98 | 0.99 | 1.00 | | 0.47 | 0.29 | 0.52 |
| fracture_calvarial | 45 | 0.78 | | 0.47 | 0.64 | | 0.97 | 0.99 | 0.99 | | 0.33 | 0.44 | 0.48 |
| fracture_skull_base | 32 | 0.47 | | 0.24 | 0.37 | | 0.99 | 0.99 | 0.99 | | 0.37 | 0.55 | 0.56 |
| fungal_sinusitis | 222 | 0.67 | | 0.43 | 0.65 | | 0.98 | 0.98 | 0.98 | | 0.71 | 0.50 | 0.57 |
| globe_shape_abnormality | 69 | 0.42 | | 0.39 | 0.61 | | 1.00 | 0.99 | 0.99 | | 0.67 | 0.84 | 0.84 |
| haemorrhagic_contusion | 81 | 0.84 | | 0.49 | 0.76 | | 0.97 | 0.97 | 0.96 | | 0.42 | 0.77 | 0.71 |
| hyperdense_artery | 24 | 0.67 | | 0.15 | 0.45 | | 0.97 | 0.99 | 0.99 | | 0.16 | 0.84 | 0.83 |
| hyperostosis_frontalis | 181 | 0.63 | | 0.34 | 0.60 | | 1.00 | 0.98 | 0.98 | | 0.91 | 0.66 | 0.74 |
| hypodense_basal_ganglia | 29 | 0.62 | | 0.15 | 0.47 | | 0.99 | 1.00 | 1.00 | | 0.37 | 0.46 | 0.46 |
| hypopneumatised_mastoid | 146 | 0.42 | | 0.48 | 0.54 | | 1.00 | 0.98 | 0.98 | | 0.83 | 0.72 | 0.74 |
| hypoxic_ischaemic_encephalopathy | 42 | 0.28 | | 0.64 | 0.62 | | 1.00 | 0.99 | 0.98 | | 0.84 | 0.63 | 0.58 |
| insular_ribbon_sign | 63 | 0.57 | | 0.11 | 0.46 | | 0.99 | 1.00 | 1.00 | | 0.46 | 0.71 | 0.79 |
| intra_ocular_silicone | 41 | 0.57 | | 0.78 | 0.83 | | 1.00 | 0.99 | 0.99 | | 0.91 | 0.52 | 0.51 |
| intraaxial_lesion_calcification | 24 | 0.54 | | 0.40 | 0.55 | | 0.98 | 0.99 | 0.99 | | 0.16 | 0.29 | 0.32 |
| intraaxial_lesion_complex_cyst | 32 | 0.69 | | 0.42 | 0.57 | | 0.97 | 1.00 | 1.00 | | 0.21 | 0.82 | 0.87 |
| intraaxial_lesion_csf_cyst | 7 | 0.00 | | 0.33 | 0.21 | | 1.00 | 0.99 | 1.00 | | 0.00 | 0.48 | 0.54 |
| intraaxial_lesion_haemorrhage | 39 | 0.87 | | 0.42 | 0.63 | | 0.94 | 0.99 | 0.99 | | 0.18 | 0.52 | 0.53 |
| intraaxial_lesion_hyperdense | 44 | 0.59 | | 0.40 | 0.51 | | 0.96 | 0.99 | 0.99 | | 0.19 | 0.76 | 0.77 |
| intraaxial_lesion_hypodense | 25 | 0.64 | | 0.16 | 0.37 | | 0.94 | 0.99 | 0.99 | | 0.09 | 0.52 | 0.58 |
| intraaxial_lesion_isodense | 27 | 0.63 | | 0.16 | 0.33 | | 0.96 | 0.99 | 1.00 | | 0.14 | 0.52 | 0.60 |
| intraaxial_lesion_mixed | 49 | 0.77 | | 0.35 | 0.61 | | 0.95 | 0.99 | 0.96 | | 0.22 | 0.47 | 0.38 |
| intraventricular_haemorrhage | 102 | 0.95 | | 0.62 | 0.84 | | 0.96 | 0.99 | 0.99 | | 0.45 | 0.71 | 0.69 |
| lacunar_infarct | 424 | 0.60 | | 0.44 | 0.60 | | 0.95 | 1.00 | 1.00 | | 0.69 | 0.79 | 0.78 |
| lateral_ventricular_effacement | 335 | 0.81 | | 0.50 | 0.78 | | 0.97 | 1.00 | 1.00 | | 0.78 | 0.48 | 0.48 |
| lytic_lesion_skull_base | 83 | 0.40 | | 0.36 | 0.48 | | 0.99 | 1.00 | 1.00 | | 0.58 | 0.72 | 0.69 |
| mastoid_opacity | 444 | 0.43 | | 0.54 | 0.59 | | 0.99 | 1.00 | 1.00 | | 0.90 | 0.67 | 0.65 |
| mastoidectomy | 52 | 0.92 | | 0.61 | 0.87 | | 1.00 | 1.00 | 0.98 | | 0.98 | 0.76 | 0.54 |
| meningioma_with_hyperostosis | 36 | 0.09 | | 0.22 | 0.21 | | 1.00 | 0.99 | 0.99 | | 0.61 | 0.77 | 0.79 |
| metallic_artefact | 546 | 0.61 | | 0.54 | 0.68 | | 0.99 | 1.00 | 1.00 | | 0.95 | 0.20 | 0.17 |
| midline_shift | 214 | 0.96 | | 0.50 | 0.85 | | 0.88 | 0.99 | 0.99 | | 0.39 | 0.64 | 0.57 |
| movement_artefact | 128 | 0.24 | | 0.39 | 0.43 | | 1.00 | 1.00 | 1.00 | | 0.91 | 0.54 | 0.55 |
| mucocoele | 16 | 0.00 | | 0.36 | 0.32 | | 1.00 | 0.97 | 0.97 | | N/A | 0.64 | 0.66 |
| mucosal_thickening | 1141 | 0.42 | | 0.68 | 0.65 | | 0.98 | 0.99 | 0.99 | | 0.93 | 0.67 | 0.68 |
| neck_haematoma | 150 | 0.72 | | 0.51 | 0.71 | | 0.99 | 1.00 | 1.00 | | 0.72 | 0.66 | 0.52 |
| non_aggressive_bone_lesion | 91 | 0.02 | | 0.21 | 0.15 | | 1.00 | 0.99 | 0.98 | | 0.33 | 0.56 | 0.52 |
| non_aggressive_skin_lesion | 108 | 0.24 | | 0.39 | 0.40 | | 1.00 | 1.00 | 1.00 | | 0.79 | 0.57 | 0.59 |
| obstructive_hydrocephalus | 96 | 0.95 | | 0.26 | 0.62 | | 0.93 | 1.00 | 0.99 | | 0.30 | 0.30 | 0.38 |
| orbital_fat_stranding | 59 | 0.38 | | 0.33 | 0.51 | | 0.99 | 0.99 | 0.99 | | 0.56 | 0.47 | 0.45 |
| orbital_mass | 41 | 0.25 | | 0.37 | 0.43 | | 0.99 | 0.99 | 0.99 | | 0.29 | 0.78 | 0.84 |
| orbital_prosthesis | 52 | 0.84 | | 0.85 | 0.92 | | 0.99 | 0.99 | 0.99 | | 0.60 | 0.28 | 0.37 |
| osteoma | 95 | 0.32 | | 0.29 | 0.38 | | 1.00 | 0.97 | 0.98 | | 0.79 | 0.53 | 0.69 |
| parotid_lesion | 47 | 0.09 | | 0.28 | 0.26 | | 1.00 | 0.98 | 1.00 | | 0.80 | 0.55 | 0.77 |
| perimesencephalic_aneurysmal_ subarachnoid_haemorrhage | 66 | 0.94 | | 0.44 | 0.68 | | 0.96 | 1.00 | 0.99 | | 0.35 | 0.41 | 0.56 |
| peripheral_acute_infarct | 64 | 0.00 | | 0.03 | 0.02 | | 1.00 | 1.00 | 1.00 | | N/A | 0.76 | 0.83 |
| perivascular_spaces | 338 | 0.15 | | 0.22 | 0.23 | | 1.00 | 0.98 | 0.96 | | 0.81 | 0.72 | 0.64 |
| petrous_bone_fracture | 57 | 0.51 | | 0.52 | 0.68 | | 1.00 | 0.99 | 0.99 | | 0.72 | 0.37 | 0.31 |
| pineal_mass | 22 | 0.27 | | 0.27 | 0.32 | | 0.98 | 1.00 | 0.99 | | 0.09 | 0.59 | 0.49 |
| resection_cavity | 85 | 0.44 | | 0.38 | 0.46 | | 0.99 | 0.99 | 1.00 | | 0.70 | 0.13 | 0.14 |
| scalp_haematoma | 397 | 0.73 | | 0.46 | 0.67 | | 0.96 | 0.98 | 0.97 | | 0.75 | 0.34 | 0.30 |
| sellar_haematoma | 43 | 0.14 | | 0.48 | 0.52 | | 1.00 | 0.99 | 0.98 | | 0.86 | 0.34 | 0.29 |
| sellar_lesion | *87* | 0.74 | | 0.57 | 0.70 | | 0.99 | 0.98 | 0.97 | | 0.71 | 0.12 | 0.13 |
| simple_pineal_cyst | 58 | 0.28 | | 0.24 | 0.44 | | 1.00 | 0.99 | 0.98 | | 0.89 | 0.18 | 0.18 |
| sino_nasal_surgery | 192 | 0.43 | | 0.28 | 0.49 | | 0.98 | 0.99 | 0.97 | | 0.57 | 0.37 | 0.33 |
| sinus_fluid_level | 306 | 0.86 | | 0.56 | 0.76 | | 0.87 | 0.95 | 0.95 | | 0.44 | 0.50 | 0.51 |
| sinus_lesion | 66 | 0.35 | | 0.37 | 0.48 | | 0.99 | 1.00 | 1.00 | | 0.44 | 0.00 | 0.00 |
| skull_vault_haemangioma | 64 | 0.00 | | 0.23 | 0.16 | | 1.00 | 1.00 | 0.99 | | N/A | 0.84 | 0.73 |
| small_vessel_disease | 1081 | 0.79 | | 0.55 | 0.79 | | 0.90 | 0.99 | 0.99 | | 0.83 | 0.83 | 0.73 |
| soft_tissue_mass_neck | 55 | 0.27 | | 0.32 | 0.43 | | 1.00 | 0.92 | 0.92 | | 0.52 | 0.55 | 0.60 |
| striatocapsular_slit_like_chronic_hemorrhage | 38 | 0.03 | | 0.22 | 0.15 | | 1.00 | 0.99 | 0.97 | | 1.00 | 0.89 | 0.77 |
| subacute_intraparenchymal_haemorrhage | 41 | 0.07 | | 0.24 | 0.25 | | 1.00 | 0.99 | 0.99 | | 0.50 | 0.61 | 0.65 |
| subacute_subdural_haematoma | 79 | 0.74 | | 0.52 | 0.66 | | 0.97 | 1.00 | 1.00 | | 0.42 | 0.68 | 0.76 |
| subcutaneous_emphysema | 167 | 0.60 | | 0.33 | 0.63 | | 0.97 | 0.92 | 0.96 | | 0.52 | 0.67 | 0.76 |
| subependymal_calcification | 66 | 0.02 | | 0.41 | 0.39 | | 1.00 | 1.00 | 1.00 | | 1.00 | 0.82 | 0.89 |
| sulcal_effacement | 679 | 0.96 | | 0.39 | 0.82 | | 0.80 | 1.00 | 1.00 | | 0.59 | 0.59 | 0.62 |
| third_ventricular_effacement | 132 | 0.65 | | 0.42 | 0.67 | | 0.99 | 0.99 | 0.99 | | 0.78 | 0.94 | 0.94 |
| tmj_arthritis | 476 | 0.20 | | 0.49 | 0.44 | | 0.99 | 0.99 | 0.93 | | 0.79 | 0.88 | 0.54 |
| tmj_dislocation | 46 | 0.15 | | 0.47 | 0.40 | | 1.00 | 0.99 | 1.00 | | 0.59 | 0.79 | 0.87 |
| tonsillar_herniation | 38 | 0.83 | | 0.30 | 0.67 | | 0.99 | 0.98 | 0.99 | | 0.44 | 0.21 | 0.32 |
| transependymal_oedema | 24 | 0.61 | | 0.31 | 0.50 | | 0.99 | 0.87 | 0.90 | | 0.35 | 0.79 | 0.82 |
| transphenoidal_surgery | 35 | 0.41 | | 0.57 | 0.62 | | 1.00 | 0.99 | 0.99 | | 0.72 | 0.78 | 0.74 |
| tympanic_cavity_erosion | 21 | 0.00 | | 0.30 | 0.23 | | 1.00 | 0.99 | 0.99 | | N/A | 0.56 | 0.62 |
| tympanic_cavity_opacity | 171 | 0.83 | | 0.55 | 0.81 | | 0.99 | 0.98 | 0.99 | | 0.84 | 0.35 | 0.36 |
| uncal_herniation | 68 | 0.92 | | 0.39 | 0.79 | | 0.97 | 0.98 | 0.99 | | 0.42 | 0.52 | 0.60 |
| vascular_clips | 54 | 0.66 | | 0.61 | 0.74 | | 0.99 | 0.99 | 1.00 | | 0.56 | 0.26 | 0.33 |
| vasogenic_oedema | 299 | 0.97 | | 0.66 | 0.86 | | 0.87 | 0.99 | 0.96 | | 0.47 | 0.54 | 0.37 |
| ventricular_cyst | 29 | 0.07 | | 0.24 | 0.28 | | 1.00 | 1.00 | 0.99 | | 0.14 | 0.05 | 0.08 |
| ventricular_mass | 29 | 0.07 | | 0.30 | 0.25 | | 1.00 | 1.00 | 1.00 | | 0.66 | 0.44 | 0.50 |
| vitreous_haemorrrhage | 26 | 0.48 | | 0.70 | 0.75 | | 1.00 | 0.99 | 0.99 | | 0.46 | 0.54 | 0.55 |
| vp_shunt | 193 | 0.92 | | 0.93 | 0.93 | | 1.00 | 1.00 | 0.99 | | 0.98 | 0.77 | 0.74 |
| watershed_acute_infarct | 111 | 0.45 | | 0.15 | 0.37 | | 0.99 | 0.99 | 0.99 | | 0.61 | 0.52 | 0.45 |

Supplementary Table 7: CTB model beta values.

| *Clinical finding* | *Beta value* |
| --- | --- |
| *Abnormal_prominent_vessels* | *3.5* |
| *Acute_haemorrhagic_infarct* | *2.75* |
| *Acute_infarct* | *4.25* |
| *Acute_infarct_petechial* | *2.5* |
| *Acute_intraparenchymal_haemorrhage* | *5* |
| *Acute_lacunar_infarct* | *2.75* |
| *Acute_on_chronic_subdural_haematoma* | *3.25* |
| *Acute_subdural_haematoma* | *3.5* |
| *Agenesis_corpus_callosum* | *0.875* |
| *Aggressive_lytic_lesion_calvarial* | *3.5* |
| *Aggressive_meningeal_thickening* | *1.9375* |
| *Aggressive_mixed_lesion_calvarial* | *3.5* |
| *Aggressive_skin_lesion* | *2* |
| *Aneurysm* | *3.5* |
| *Aneurysm_coils* | *1* |
| *Basal_cistern_effacement* | *1.75* |
| *Basal_ganglia_calcification* | *0.625* |
| *Beam_hardening_artefact* | *0.375* |
| *Benign_meningeal_thickening* | *0.5625* |
| *Brainstem_infarct* | *2.5* |
| *Calvarial_thickening* | *0.5625* |
| *Cavum_septum_pellucidum* | *0.3625* |
| *Cerebellar_atrophy* | *0.5* |
| *Cerebellar_infarct* | *2.75* |
| *Cerebral_atrophy* | *0.4375* |
| *Cerebral_convexity_subarachnoid_haemorrhage* | *5* |
| *Chiari_1* | *0.875* |
| *Chronic_subdural_haematoma* | *2* |
| *Cochlear_implant* | *1* |
| *Colloid_cyst* | *6* |
| *Colpocephaly* | *0.875* |
| *Communicating_hydrocephalus* | *1* |
| *Confluent_white_matter_hypodensities* | *0.5625* |
| *Cortical_laminar_necrosis* | *0.5* |
| *Cortical_leptomeningeal_calcification* | *0.5625* |
| *Craniotomy* | *1* |
| *Craniotomy_collection* | *1* |
| *Dbs_electrodes* | *1* |
| *Deep_white_gray_matter_infarct* | *0.8125* |
| *Demyelination* | *0.5625* |
| *Dilated_superior_opthalmic_vein* | *0.875* |
| *Dural_calcification* | *0.6875* |
| *Empty_sella* | *1* |
| *Encephalomalacia* | *0.875* |
| *Entrapment_lateral_ventricle* | *0.875* |
| *Evd* | *1* |
| *Exophthalmos* | *0.875* |
| *Expanded_pituitary_fossa* | *1.1875* |
| *Extra_axial_collection_air* | *3.25* |
| *Extra_axial_collection_csf* | *0.875* |
| *Extra_axial_mass_calcific_density_non_aggressive* | *1.375* |
| *Extra_axial_mass_fat_density_non_aggressive* | *1.5* |
| *Extra_axial_mass_soft_tissue_density_aggressive* | *3* |
| *Extra_axial_mass_soft_tissue_density_non_aggressive* | *2.25* |
| *Extracranial_herniation* | *1* |
| *Extradural_haematoma* | *3.5* |
| *Extraocular_muscle_enlargement* | *2.75* |
| *Facial_bone_fracture* | *1.75* |
| *Facial_bony_destruction* | *2* |
| *Fibrous_dysplasia* | *0.625* |
| *Focal_calcification* | *2.5* |
| *Foreign_body_neck* | *3* |
| *Foreign_body_orbit* | *3.75* |
| *Foreign_body_scalp* | *3* |
| *Fourth_ventricular_effacement* | *2* |
| *Fracture_calvarial* | *3* |
| *Fracture_skull_base* | *3.5* |
| *Fungal_sinusitis* | *0.675* |
| *Globe_shape_abnormality* | *0.8125* |
| *Haemorrhagic_contusion* | *2.75* |
| *Hyperdense_artery* | *4.5* |
| *Hyperostosis_frontalis* | *0.28125* |
| *Hypodense_basal_ganglia* | *2.5* |
| *Hypopneumatised_mastoid* | *0.375* |
| *Hypoxic_ischaemic_encephalopathy* | *3.5* |
| *Insular_ribbon_sign* | *2.75* |
| *Intra_ocular_silicone* | *0.8125* |
| *Intraaxial_lesion_calcification* | *3* |
| *Intraaxial_lesion_complex_cyst* | *2.75* |
| *Intraaxial_lesion_csf_cyst* | *2.5* |
| *Intraaxial_lesion_haemorrhage* | *3.5* |
| *Intraaxial_lesion_hyperdense* | *3* |
| *Intraaxial_lesion_hypodense* | *3* |
| *Intraaxial_lesion_isodense* | *3* |
| *Intraaxial_lesion_mixed* | *3* |
| *Intraventricular_haemorrhage* | *5* |
| *Lacunar_infarct* | *0.625* |
| *Lateral_ventricular_effacement* | *1* |
| *Lytic_lesion_skull_base* | *3.75* |
| *Mastoid_opacity* | *0.28125* |
| *Mastoidectomy* | *1* |
| *Meningioma_with_hyperostosis* | *2.25* |
| *Metallic_artefact* | *0.375* |
| *Midline_shift* | *3.75* |
| *Movement_artefact* | *0.375* |
| *Mucocoele* | *0.875* |
| *Mucosal_thickening* | *0.15* |
| *Neck_haematoma* | *1* |
| *Non_aggressive_bone_lesion* | *0.30625* |
| *Non_aggressive_skin_lesion* | *0.5625* |
| *Obstructive_hydrocephalus* | *4* |
| *Orbital_fat_stranding* | *3.25* |
| *Orbital_mass* | *3.75* |
| *Orbital_prosthesis* | *0.6875* |
| *Osteoma* | *0.40625* |
| *Parotid_lesion* | *1.5* |
| *Perimesencephalic_aneurysmal_subarachnoid_haemorrhage* | *5* |
| *Peripheral_acute_infarct* | *2.5* |
| *Perivascular_spaces* | *0.5625* |
| *Petrous_bone_fracture* | *3* |
| *Pineal_mass* | *0.8* |
| *Resection_cavity* | *1* |
| *Scalp_haematoma* | *1* |
| *Sellar_haematoma* | *3.75* |
| *Sellar_lesion* | *2.75* |
| *Simple_pineal_cyst* | *0.75* |
| *Sino_nasal_surgery* | *1* |
| *Sinus_fluid_level* | *1.875* |
| *Sinus_lesion* | *1.75* |
| *Skull_vault_haemangioma* | *0.375* |
| *Small_vessel_disease* | *0.5* |
| *Soft_tissue_mass_neck* | *1.25* |
| *Striatocapsular_slit-like_chronic_hemorrhage* | *0.625* |
| *Subacute_intraparenchymal_haemorrhage* | *4.5* |
| *Subacute_subdural_haematoma* | *2.75* |
| *Subcutaneous_emphysema* | *1.125* |
| *Subependymal_calcification* | *0.5* |
| *Sulcal_effacement* | *3.25* |
| *Third_ventricular_effacement* | *1* |
| *Tmj_arthritis* | *0.30625* |
| *Tmj_dislocation* | *2.25* |
| *Tonsillar_herniation* | *3.75* |
| *Transependymal_oedema* | *2.5* |
| *Transphenoidal_surgery* | *1* |
| *Tympanic_cavity_erosion* | *1.125* |
| *Tympanic_cavity_opacity* | *1* |
| *Uncal_herniation* | *3.75* |
| *Vascular_clips* | *1* |
| *Vasogenic_oedema* | *4* |
| *Ventricular_cyst* | *0.425* |
| *Ventricular_mass* | *3* |
| *Vitreous_haemorrrhage* | *3.25* |
| *Vp_shunt* | *1* |
| *Watershed_acute_infarct* | *2.75* |

Supplementary Table 8: Manufacturer distributions in the training and testing datasets.

| **Scanner Manufacturer** | **Training Dataset Count (%)** | **Test Dataset Count (%)** |
| --- | --- | --- |
| Toshiba | 77,946 (33.95) | 1,271 (44.63) |
| Siemens | 60,091 (26.17) | 406 (14.26) |
| GE medical systems | 59,987 (26.13) | 738 (25.91) |
| Philips | 29,840 (13) | 422 (14.82) |
| Canon Medical Systems | 1,688 (0.74) | 11 (0.39) |
| Siemens Healthineers | 13 (0.01) | 0 (0) |
| PNMS | 9 (0) | 0 (0) |
| Mobius imaging, LLC | 1 (0) | 0 (0) |


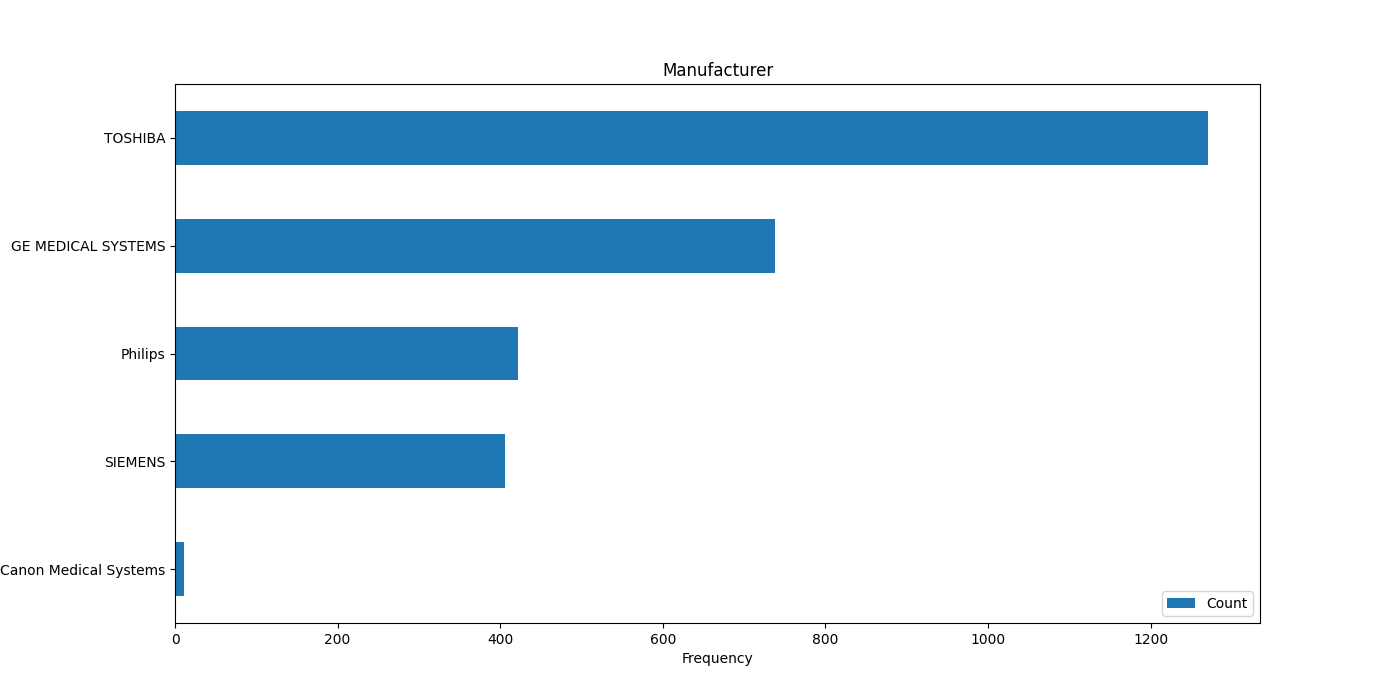


*Figure 1: Test set manufacturer distribution.*

*
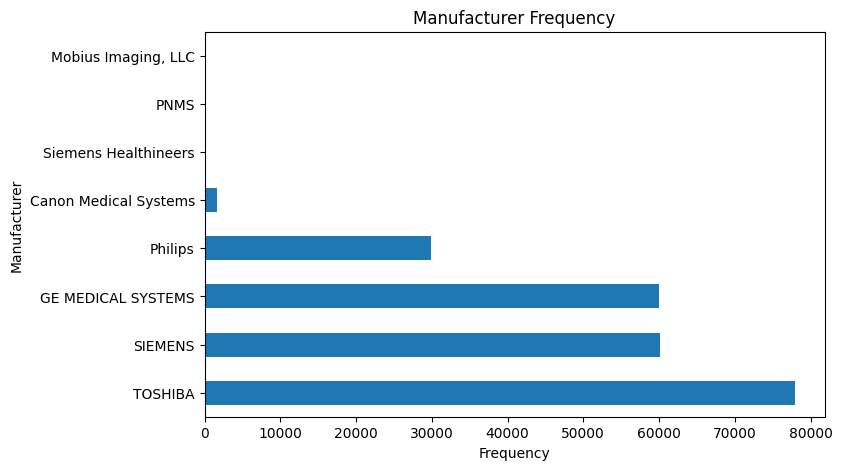
*

*Figure 2: Training set manufacturer distribution.*
